# Brain Frailty and Functional Outcomes After Thrombolysis for Acute Ischemic Stroke

**DOI:** 10.1001/jamanetworkopen.2025.34941

**Published:** 2025-09-30

**Authors:** Spencer P. Loewen, Nishita Singh, Ibrahim Alhabli, Fouzi Bala, Brian Buck, Faysal Benali, William Betzner, Kaden Lam, Luciana Catanese, Aleksander Tkach, Dar Dowlatshahi, Federico Carpani, Thalia S. Field, Gary Hunter, Houman Khosravani, Aleksandra Pikula, Michel Shamy, Tolulope T. Sajobi, Mohammed Almekhlafi, Rick Swartz, Bijoy Menon, Mahesh Kate, Aravind Ganesh

**Affiliations:** 1 Calgary Stroke Program, Cumming School of Medicine, University of Calgary, Calgary, Alberta, Canada; 2 Neurology Section, Department of Internal Medicine, Rady Faculty of Health Sciences, University of Manitoba, Winnipeg, Canada; 3 Department of Clinical Neurosciences, the Hotchkiss Brain Institute and the O’Brien Institute for Public Health, Cumming School of Medicine, University of Calgary, Calgary, Alberta, Canada; 4 Department of Radiology, the Hotchkiss Brain Institute and the O’Brien Institute for Public Health, Cumming School of Medicine, University of Calgary, Calgary, Alberta, Canada; 5 Department of Community Health Sciences, the Hotchkiss Brain Institute and the O’Brien Institute for Public Health, Cumming School of Medicine, University of Calgary, Calgary, Alberta, Canada; 6 Diagnostic and Interventional Neuroradiology Department, University Hospital of Tours, Tours, France; 7 Division of Neurology, Department of Medicine, University of Alberta, Edmonton, Canada; 8 Department of Radiology, AZ Vesalius, Tongeren, Belgium; 9 Graduate Science Education, Cumming School of Medicine, University of Calgary, Calgary, Alberta, Canada; 10 Department of Medicine, McMaster University and Population Health Research Institute, Hamilton, Ontario, Canada; 11 Kelowna General Hospital, Kelowna, British Columbia, Canada; 12 Department of Medicine, University of Ottawa and Ottawa Hospital Research Institute, Ottawa, Ontario, Canada; 13 Department of Neurology, Toronto Western Hospital and the University of Toronto, Toronto, Ontario, Canada; 14 Department of Neurosciences, University of British Columbia, Vancouver, British Columbia, Canada; 15 Department of Medicine, University of Saskatchewan, Saskatoon, Canada; 16 Neurology Division, Department of Medicine, Sunnybrook Health Sciences Centre, Toronto, Ontario, Canada; 17 Department of Neurology, University of Toronto, Toronto, Ontario, Canada

## Abstract

**Importance:**

The cumulative burden of chronic vascular and neurodegenerative changes contributes to brain frailty, which may reduce the brain’s capacity to recover from acute ischemic stroke (AIS). The association between brain frailty markers and poststroke outcomes after thrombolysis is unclear.

**Objective:**

To evaluate associations between brain frailty assessed on non–contrast-enhanced computed tomography (NCCT) and magnetic resonance imaging (MRI) and functional outcome in patients with AIS treated with intravenous thrombolysis.

**Design, Setting, and Participants:**

This cohort study was a post hoc analysis of the Alteplase compared to Tenecteplase (AcT) trial, an investigator-led, registry-linked, parallel-group, open-label, randomized clinical trial that enrolled patients between December 10, 2019, and January 25, 2022, at 22 primary and comprehensive stroke centers across Canada. Participants included adults 18 years or older diagnosed with ischemic stroke causing disabling neurological deficit, presenting within 4.5 hours of symptom onset, and meeting Canadian guidelines for thrombolysis. Markers of brain frailty (cortical and subcortical atrophy, white matter changes [Fazekas score, grouped as 0, 1-2, and 3-6], lacunes, chronic infarctions, and [on MRI] microbleeds, siderosis, and enlarged perivascular spaces) were retrospectively assessed while reviewers were blinded to outcome variables. Analyses were performed from July 24, 2024, to March 25, 2025.

**Exposures:**

Patients underwent baseline NCCT and were randomized to receive intravenous thrombolysis with alteplase (0.9 mg/kg) or tenecteplase (0.25 mg/kg). Some patients also received posttreatment brain MRI.

**Main Outcomes and Measures:**

The primary outcome was excellent functional outcome (modified Rankin Scale [mRS] score of 0-1) at 90 days. Secondary outcomes included 90-day ordinal mRS score (trichotomized as 0-2, 3-4, and 5-6), symptomatic intracerebral hemorrhage, and mortality. Sensitivity analyses were performed in patients with available MRI scans.

**Results:**

Among the 1568 patients (median age, 74 [IQR, 63-83] years; 817 male [52.1%]) with interpretable NCCT findings, after correcting for multiple comparisons, higher total Fazekas score of 3 to 6 compared with 0 was associated with lower odds of a 90-day mRS score of 0 to 1 (adjusted odds ratio [OR], 0.40 [95% CI, 0.24-0.65]). Total Fazekas score (adjusted common OR [ACOR], 2.80 [95% CI, 1.88-4.16]), cortical atrophy (ACOR, 2.65 [95% CI. 1.63-4.32]), and total brain frailty score (ACOR, 3.15 [95% CI, 1.87-5.33]) were each associated with worse ordinal mRS, but were not associated with safety outcomes.

**Conclusions and Relevance:**

In this cohort study of patients with AIS treated with intravenous thrombolysis, brain frailty markers—particularly white matter changes, cortical atrophy, and total brain frailty—were associated with worse outcomes. Consideration of these neuroimaging markers may better inform clinicians and patients about treatment expectations from thrombolytic therapy.

## Key Points

**Question:**

Are neuroimaging markers of brain frailty associated with functional outcomes in patients with acute ischemic stroke (AIS) treated with intravenous thrombolysis?

**Findings:**

In this cohort study of 1568 patients with AIS, the severity of white matter changes, cortical atrophy, and a total brain frailty score capturing the aggregate burden were each associated with worse functional outcomes at 90 days.

**Meaning:**

These findings suggest that brain frailty assessment on baseline neuroimaging using clinical rating scales provides prognostic information for clinicians and patients regarding functional outcomes after reperfusion therapies for AIS, including intravenous thrombolysis with alteplase or tenecteplase.

## Teaser

This cohort study explores the association of brain frailty on neuroimaging with functional and safety outcomes among participants in the Alteplase compared to Tenecteplase (AcT) who underwent intravenous thrombolysis for acute ischemic stroke.

## Introduction

Brain frailty is an emerging concept representing the cumulative burden of chronic vascular changes and neurodegeneration. Brain frailty impairs the brain’s ability to compensate and recover in response to injuries such as acute ischemic stroke (AIS), reflecting a state of diminished structural and functional brain reserve.^1,2^

Cerebral small vessel disease (SVD) and brain atrophy are key contributors to brain frailty, both of which can be assessed on conventional neuroimaging. Imaging markers of brain frailty include conventional SVD markers, as well as chronic nonlacunar infarctions, and cerebral atrophy encompassing both cortical and subcortical regions.^3^ These are commonly identified on non–contrast-enhanced computed tomography (NCCT) or magnetic resonance imaging (MRI).^4^ Composite SVD scores have been developed by combining individual markers to capture the overall burden of disease. Similar composite scores have also incorporated atrophy markers to assess total brain frailty.^2,5,6,7^ The association between brain frailty markers and poststroke outcomes after thrombolysis, however, is unclear.

Thrombolytic therapy is a cornerstone of AIS treatment. The primary agents used include alteplase and tenecteplase, the latter having gained favor due to its ease of administration as a single bolus injection and its favorable pharmacokinetic profile. Evidence from the Alteplase Compared to Tenecteplase (AcT) randomized clinical trial demonstrated that tenecteplase is noninferior to alteplase in terms of efficacy.^8^ Understanding how neuroimaging markers of brain frailty influence outcomes following treatment with these thrombolytic agents is crucial, as it may inform clinical decision-making and treatment expectations. Therefore, we explored the association between the burden of brain frailty in neuroimaging and key efficacy and safety outcomes in patients following intravenous thrombolysis for AIS.

## Methods

AcT was an investigator-led, multicenter, registry-linked, parallel-group, open-label, randomized clinical trial that enrolled patients from 22 primary and comprehensive stroke centers across Canada.^9^ Health Canada and the ethics boards at each participating center approved the trial. The trial used deferred consent procedures wherever approved by local research ethics boards, and written or electronic informed consent was obtained from all patients or their legal representatives. Eligible participants were enrolled between December 10, 2019, and January 25, 2022, and included adults 18 years or older diagnosed with ischemic stroke causing disabling neurological deficit, presenting within 4.5 hours of symptom onset, and meeting Canadian guidelines for thrombolysis. Data were analyzed from July 24, 2024, to March 25, 2025. Participants were randomized to receive either intravenous alteplase (0.9 mg/kg) or tenecteplase (0.25 mg/kg). The trial methodology has been previously published.^8,10^ This post hoc analysis of the AcT study followed the Reporting of Observational Studies in Epidemiology (STROBE) reporting guideline for cohort studies.

### Image Analysis

Patients were enrolled between December 10, 2019, and January 25, 2022. Baseline (NCCT) and follow-up (NCCT and/or MRI brain) imaging were reviewed by the imaging core laboratory (including N.S., I.A., F. Bala, and F. Benali, with 4-10 years of experience, trained and supervised by A.G.), blinded to treatment allocation and clinical outcomes. Reviewers were also blinded to follow-up imaging while reading baseline images. Scans were deemed uninterpretable if they contained significant motion artifacts, extensive beam hardening, or extensive edema.

Individual brain frailty measures were evaluated on all available baseline NCCT scans and additionally on any MRI scans performed during follow-up. The eMethods and eTable 1 in Supplement 1 show detailed definitions and descriptions of the analyses of neuroimaging markers. Briefly, cortical atrophy was assessed using the Global Cortical Atrophy (GCA) scale.^11^ Subcortical atrophy was assessed by calculating the ratio of intercaudate distance to inner-table width (CC:IT ratio). Chronic infarctions and lacunes were characterized based on location and size,^4^ and additionally combined under the umbrella of chronic vascular lesions. White matter change (WMC) was assessed using the Fazekas scale,^12^ with periventricular and deep WMCs each graded from 0 to 3 and then summated to give a total Fazekas score ranging from 0 to 6, as done in prior studies.^13^ Follow-up MRI scans were further assessed for cerebral microbleeds (CMB), enlarged perivascular spaces (EPVS), and cortical superficial siderosis (CSS). A total SVD score was calculated from MRI-based markers whereby 1 point each was added for severe WMC (total Fazekas score, 3-6), moderate to severe EPVS in basal ganglia (EPVS score, 2-4), any lacunes, and any CMB (maximum, 4 of 4).^5^ Total brain frailty scores were calculated for both CT-based markers and MRI-based markers whereby 1 point each was added for severe WMC (total Fazekas score, 3-6), severe cortical (GCA, score 2 or 3) or subcortical atrophy (highest CC:IT ratio quartile), and any vascular lesions (maximum: 3 of 3).^2^ Interrater and intrarater agreement were assessed using the Gwet agreement coefficient owing to a large proportion of zero or mild values, to avoid the κ paradox.^14,15^

### Outcomes

The primary outcome was excellent functional outcome (modified Rankin Scale [mRS] score, 0-1) at 90 days. The secondary outcome was 90-day ordinal mRS score that was trichotomized as 0 to 2, 3 to 4, and 5 to 6. The mRS is a categorical scale ranging from 0 to 6 for functional neurological outcomes, with 0 indicating no neurological symptoms and 6 indicating death. Safety outcomes were symptomatic intracerebral hemorrhage (ICH; any ICH responsible for worsening of neurological condition), radiographic ICH (any ICH visible on NCCT or MRI), severity of ICH (based on the Heidelberg Bleeding Classification),^16^ and 90-day all-cause mortality (mRS score 6).

### Statistical Analysis

Baseline clinical and imaging characteristics were compared across each total brain frailty score category (0, 1, 2, or 3). Categorical variables were summarized as counts and percentages, and statistical comparisons were conducted using the χ^2^ test. For continuous variables, data were summarized as medians with IQR and compared using the Kruskal-Wallis test.

We used mixed-effects binary logistic regression to examine associations between imaging markers and odds of achieving excellent functional outcome (mRS score 0-1) and mixed-effects ordinal logistic regression to assess association with ordinal mRS score. Mixed-effects logistic regression was also used for the association between imaging markers and safety outcomes (symptomatic ICH, radiographic ICH, ICH severity, and mortality). Unadjusted and adjusted odds ratios (ORs) with 95% CIs were obtained for each outcome of interest. The robustness of association between each brain frailty marker and outcomes of interest was examined both in terms of the effect size of the association as well as the consistency of the association across sensitivity analyses. Adjustments were made for age, sex, pretreatment National Institutes of Health Stroke Scale (NIHSS) score, and stroke symptom onset-to-needle time as fixed-effects variables, and participating site as a random-effects variable. Interactions between each brain frailty marker and thrombolytic type were tested using multiplicative terms. The area under the receiver operating characteristics curve (AUROC) was calculated for each model for the primary outcome (mRS score 0-1). The Brant test was performed to test for proportional odds assumptions for the secondary outcome (ordinal mRS score) using standard ordinal logistic regression models (eTables 2 and 3 in Supplement 1). Sensitivity analyses were performed to examine associations between imaging markers and outcomes of interest assessed on NCCT compared with those assessed on MRI. An additional sensitivity analysis was performed for total brain frailty score adjusted for endovascular thrombectomy (EVT), hypertension, and diabetes. Given that CC:IT ratios can only range from 0 to 1, with much smaller ranges seen in typical populations, conventional ORs per 1-unit increase in CC:IT ratio would be clinically uninterpretable. Therefore, we multiplied the CC:IT ratio by 100 to simplify resulting ORs (ie, they would represent the odds change with each increment of 0.01 in the CC:IT ratio). All statistical tests were 2 sided, with significance defined as *P* < .05. A Hochberg step-up procedure was used to control the familywise error rate and correct for multiple comparisons. A total of 247 tests were performed (α = .05) for a calculated adjusted significance level of *P* < .001. Statistical analyses were performed using Stata/MP, version 16.1 (StataCorp LLC).

## Results

Among 1577 patients in the intention-to-treat population in the AcT trial, 1568 (99.4%) had interpretable baseline NCCT scans and were included in this post hoc cohort analysis. The median age of these participants was 74 (IQR, 63-83) years; 751 (47.9%) were female and 817 (52.1%) were male. Baseline characteristics of patients across each total brain frailty score category are summarized in the Table. Brain frailty scores were 0 (no frailty) in 665 participants (42.4%), 1 (mild) in 486 participants (31.0%), 2 (moderate) in 289 participants (18.4%), and 3 (severe) in 128 participants (8.2%). More severe brain frailty was associated with older age, female sex, worse baseline NIHSS score, and better range of baseline Alberta Stroke Program Early CT Score (Table). For sensitivity analyses using MRI, 495 patients had available scans for analyzing brain frailty measures, for all of whom brain frailty scores could be calculated (characteristics in eTable 4 in Supplement 1). Baseline characteristics of patients who underwent follow-up MRI compared with those who did not are summarized in eTable 5 in Supplement 1. Fazekas score, GCA score, CC:IT ratio, lacunes, and chronic infarcts each had substantial to almost-perfect interrater and intrarater agreement (75%-97% interrater agreement; Gwet agreement coefficient 1: 0.63-0.96 for interrater and 0.61-0.92 for intrarater) (eTable 6 in Supplement 1).

**Table.** Baseline Characteristics of Participants Stratified by Total Brain Frailty Score Assessed on Non–Contrast-Enhanced Computed Tomography

Characteristic	Brain frailty score					*P* value
	All (N = 1568)	0 (n = 665)	1 (n = 486)	2 (n = 289)	3 (n = 128)	
Age, median (IQR), y	74 (63-83)	66 (56-76)	75 (66-84)	81 (74-87)	83 (77-89)	<.001
Sex, No. (%)						
Female	751 (47.9)	284 (42.7)	243 (50.0)	153 (52.9)	71 (55.5)	.003
Male	817 (52.1)	381 (57.3)	243 (50.0)	136 (47.1)	57 (44.5)	
Baseline NIHSS score, median (IQR)^a^	9 (6-16)	9 (5-16)	10 (6-17)	9 (6-17)	11 (6-16)	.008
Baseline ASPECTS, median (IQR)	9 (8-10)	9 (8-10)	9 (8-10)	10 (8-10)	9 (9-10)	<.001
Stroke symptom onset to needle time, median (IQR), min^b^	36 (28-49)	36 (28-49)	36 (27-48)	36 (28-48)	39 (32-52)	.07
Comorbidities, No. (%)^c^						
Hypertension	715 (49.1)	299 (49.3)	221 (48.8)	143 (52.6)	52 (44.1)	.47
Diabetes	274 (18.8)	109 (18.0)	77 (17.0)	65 (23.9)	23 (19.5)	.12
Atrial fibrillation	191 (13.1)	73 (12.1)	62 (13.7)	45 (16.5)	11 (9.3)	.17
Dyslipidemia	76 (5.2)	30 (5.0)	28 (6.2)	14 (5.2)	4 (3.4)	.63
Coronary artery disease	27 (1.9)	8 (1.3)	10 (2.2)	9 (3.3)	0	.09
Smoker (current or past)	17 (1.2)	9 (1.5)	2 (0.4)	3 (1.1)	3 (2.5)	.21
Previous stroke	12 (0.8)	4 (0.7)	6 (1.3)	1 (0.4)	1 (0.9)	.52
Thrombolytic treatment, No. (%)						
Tenecteplase	803 (51.2)	340 (51.1)	244 (50.2)	164 (56.7)	55 (43.0)	.07
Alteplase	765 (48.8)	325 (48.9)	242 (49.8)	125 (43.3)	73 (57.0)	
Endovascular thrombectomy	502 (32.0)	230 (34.6)	177 (36.4)	72 (24.9)	21 (16.4)	<.001
Total Fazekas score, No. (%)^d^						
0	790 (50.4)	518 (77.9)	225 (46.3)	47 (16.3)	0	<.001
1 to 2	450 (28.7)	147 (22.1)	198 (40.7)	105 (36.3)	0	
3 to 6	328 (20.9)	0	63 (13.0)	137 (47.4)	128 (100)	
Lacunes present, No. (%)	369 (23.5)	0	117 (24.1)	145 (50.2)	107 (83.6)	<.001
Lacune burden, median (IQR)^e^	0 (0-0)	0 (0-0)	0 (0-0)	1 (0-1)	1 (1-2)	<.001
≥1 Chronic infarction, No. (%)	224 (14.3)	0	88 (18.1)	91 (31.5)	45 (35.2)	<.001
Chronic infarction burden, median (IQR)^e^	0 (0-0)	0 (0-0)	0 (0-0)	0 (0-1)	0 (0-1)	<.001
Any lacune or chronic infarct, No. (%)	520 (33.2)	0	185 (38.1)	207 (71.6)	128 (100)	<.001
Global cortical atrophy score, No. (%)^f^						
0	751 (47.9)	478 (71.9)	182 (37.4)	74 (25.6)	17 (13.3)	<.001
1	625 (39.9)	187 (28.1)	243 (50.0)	131 (45.3)	64 (50.0)	
2 to 3	192 (12.2)	0	61 (12.6)	84 (29.1)	47 (36.7)	
CC:IT ratio, median (IQR)	0.13 (0.10-0.16)	0.11 (0.09-0.13)	0.14 (0.12-0.17)	0.16 (0.14-0.19)	0.18 (0.16-0.20)	<.001
Severe cortical or subcortical atrophy, No. (%)	600 (38.3)	0	238 (49.0)	234 (81.0)	128 (100)	<.001

Abbreviations: ASPECTS, Alberta Stroke Program Early Computed Tomography Score; CC:IT, intercaudate distance to inner-table width ratio; NIHSS, National Institutes of Health Stroke Scale.

^a^ Scores range from 0 to 42, with higher scores indicating greater stroke severity.

^b^ Indicates time to intravenous thrombolysis start.

^c^ Includes 1456 patients.

^d^ Higher scores indicate worse which matter changes.

^e^ Characterized by number and size.

^f^ Higher scores indicate more severe atrophy.

**Figure 1.**
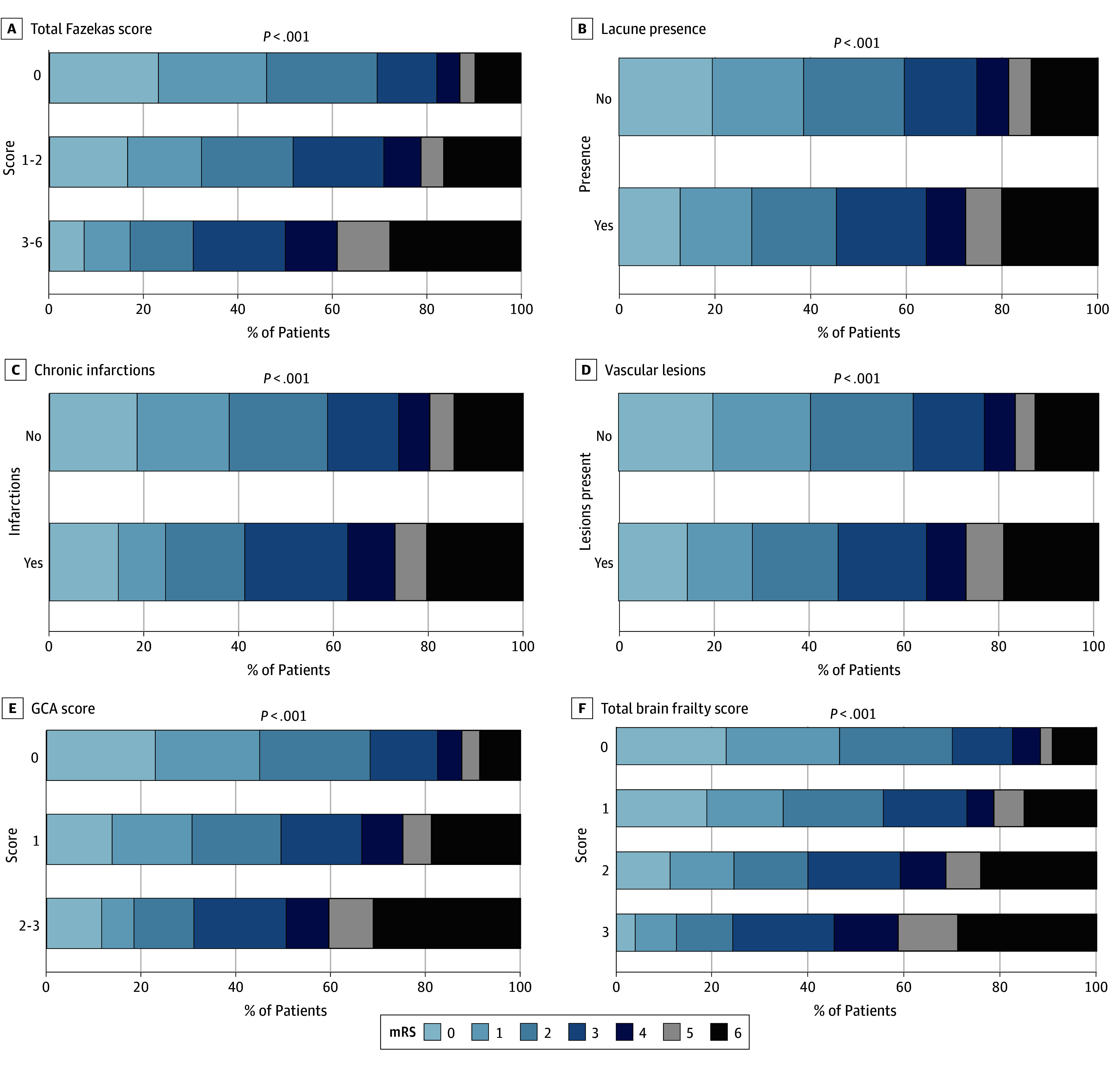
Distribution of Modified Rankin Scale (mRS) Scores at 90 Days by Brain Frailty Measures Assessed on Non–Contrast-Enhanced Computed Tomography Higher scores indicate worse findings. GCA indicates global cortical atrophy.

**Figure 2.**
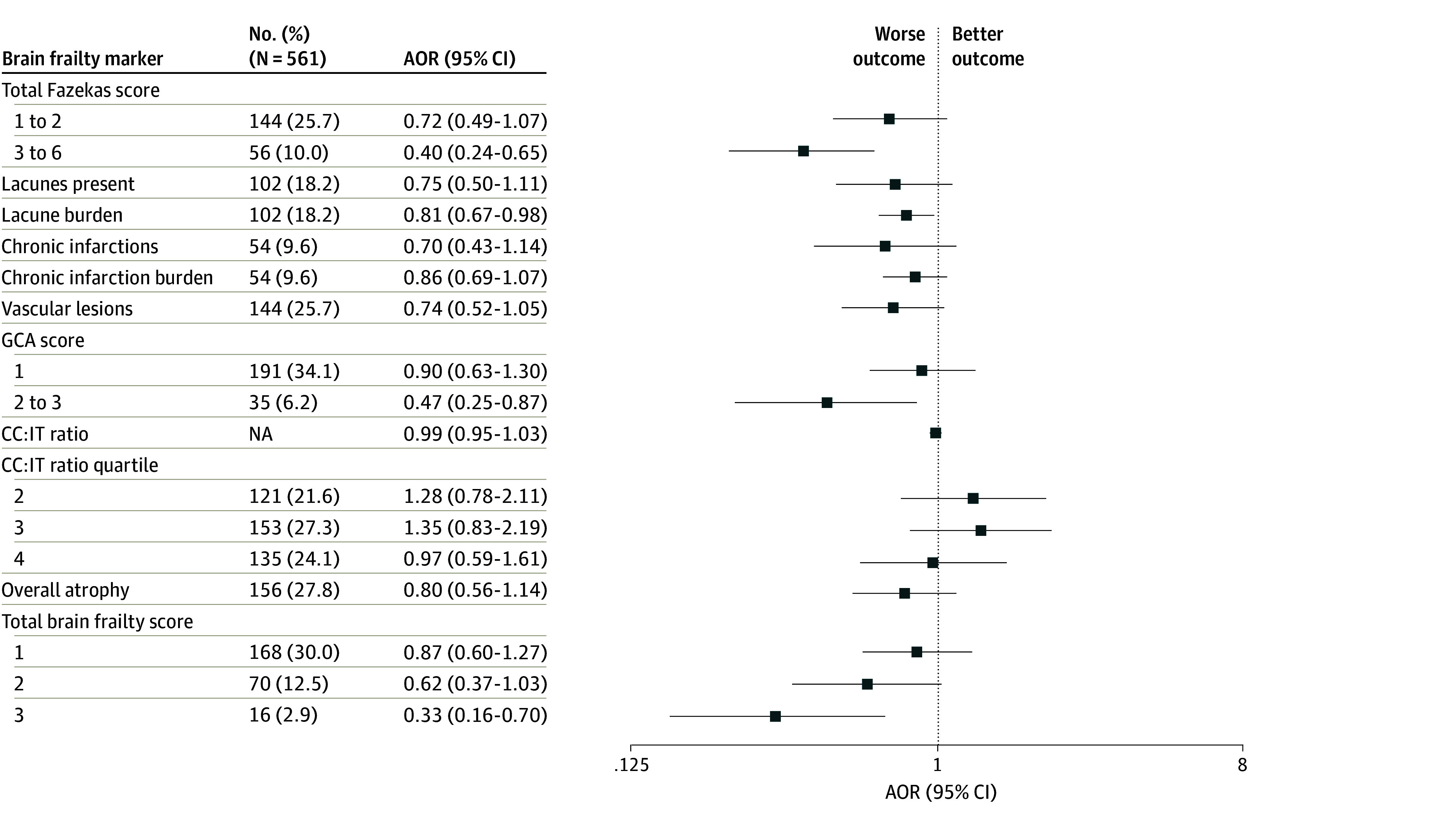
Adjusted Odds Ratios (AORs) for Modified Rankin Scale (mRS) Score of 0 to 1 by Measures of Brain Frailty Assessed on Non–Contrast-Enhanced Computed Tomography Adjustments were made for age, sex, pretreatment National Institutes of Health Stroke Scale, and stroke symptom onset-to-needle time as fixed-effects variables and participating site as a random-effects variable. CC:IT indicates intercaudate distance to inner-table width ratio; GCA, global cortical atrophy; NA, not applicable.

**Figure 3.**
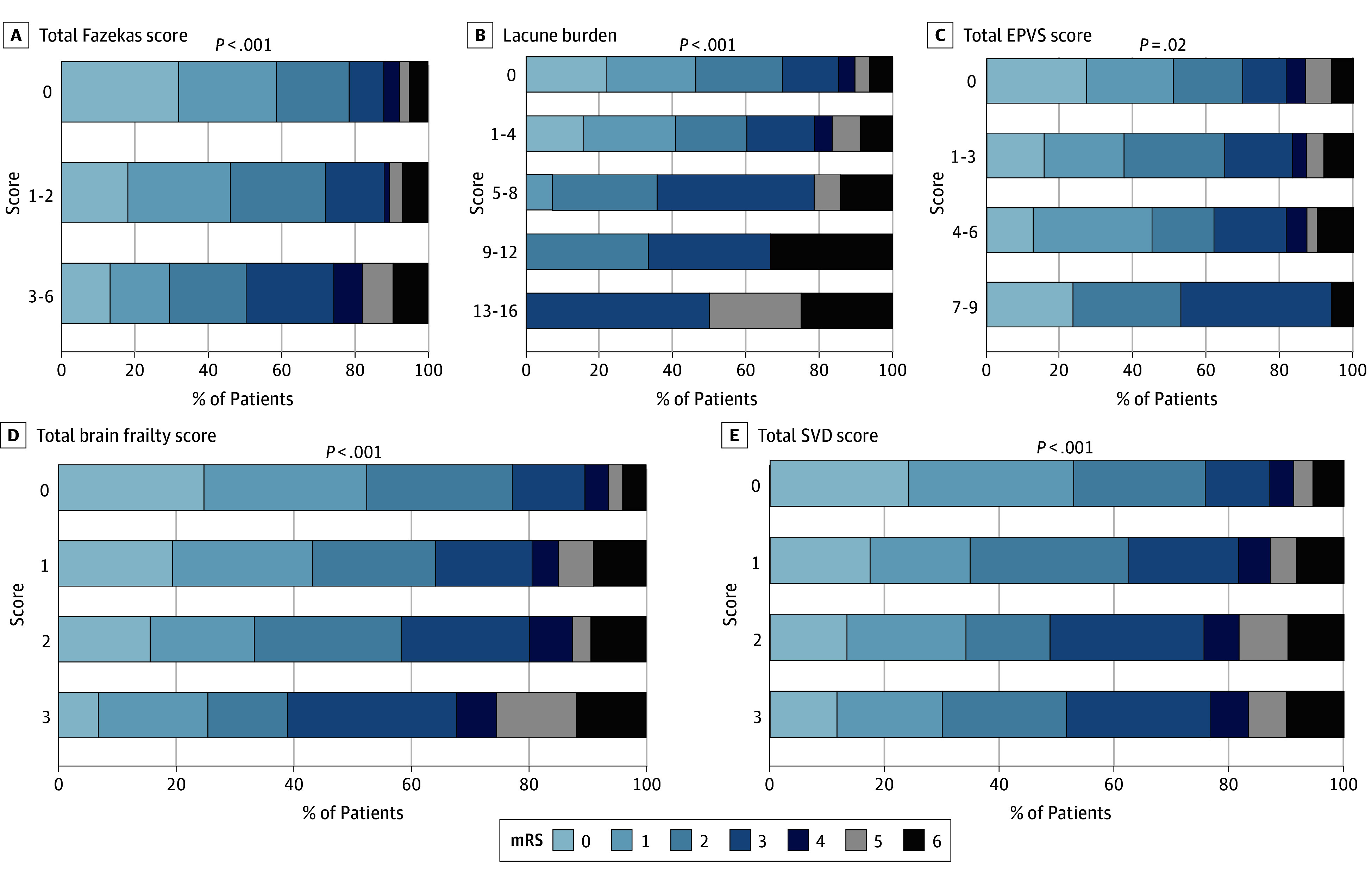
Distribution of Modified Rankin Scale (mRS) Scores at 90 Days by Brain Frailty Measures Assessed on Magnetic Resonance Imaging Higher scores indicate worse findings. EPVS indicates enlarged perivascular spaces; SVD, small vessel disease.

**Figure 4.**
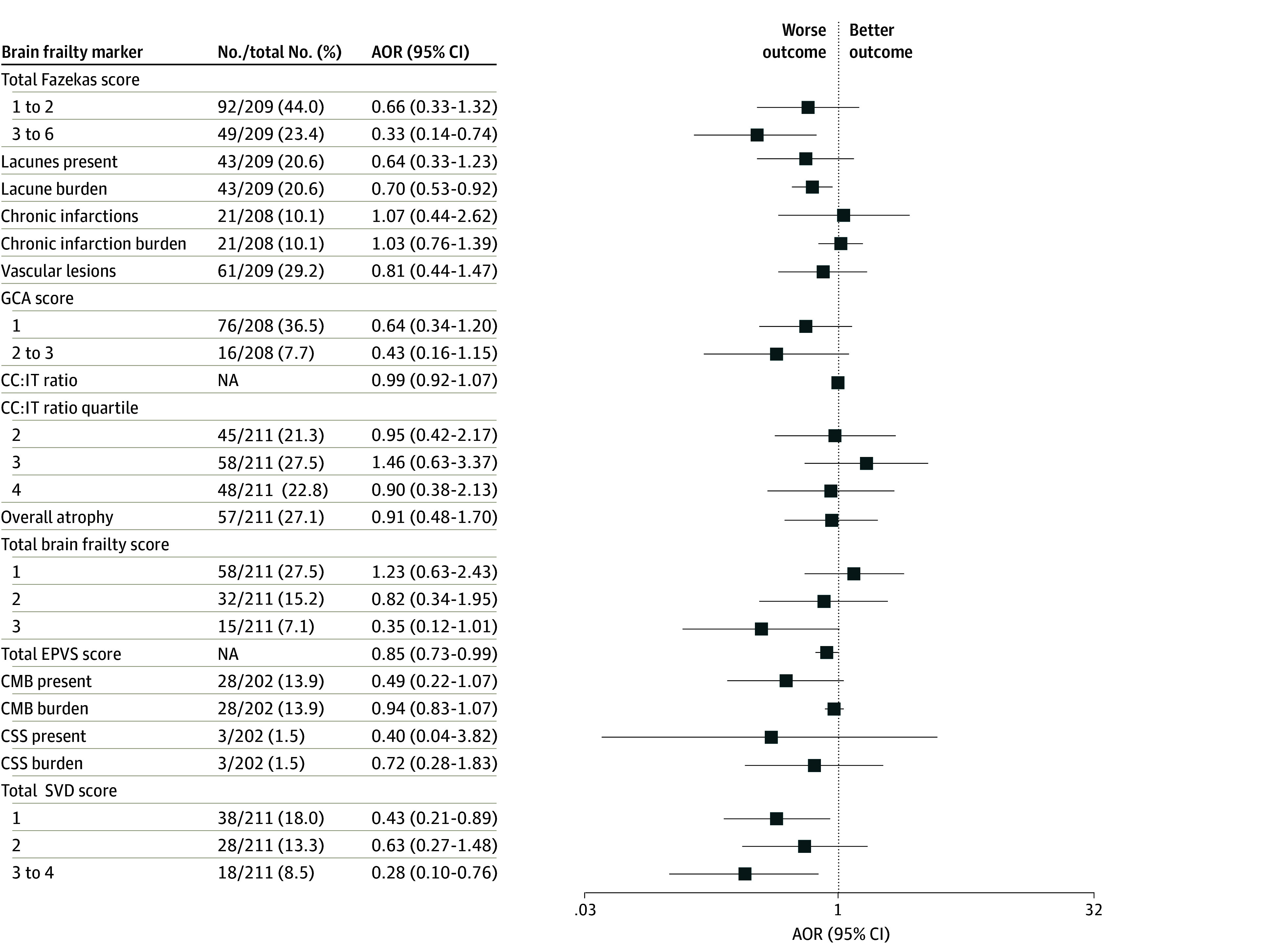
Adjusted Odds Ratios (AORs) for Modified Rankin Scale (mRS) Score of 0 to 1 by Measures of Brain Frailty Assessed on Magnetic Resonance Imaging Adjustments were made for age, sex, pretreatment National Institutes of Health Stroke Scale, and stroke symptom onset-to-needle time as fixed-effects variables and participating site as a random-effects variable. CC:IT indicates intercaudate distance to inner-table width ratio; CMB, cerebral microbleeds; CSS, cortical superficial siderosis; EPVS, enlarged perivascular spaces; GCA, global cortical atrophy; NA, not applicable; SVD, small vessel disease.

All brain frailty markers examined were negatively associated with 90-day mRS scores of 0 to 1 in unadjusted analyses, and all markers except chronic infarction burden remained significant with the Hochberg correction (eTable 7 in Supplement 1). After adjustment for age, sex, pretreatment NIHSS, and stroke symptom onset-to-needle time as fixed-effects and participating site as random-effects variables, higher total Fazekas score of 3 to 6 compared with 0 (adjusted OR [AOR], 0.40 [95% CI, 0.24-0.65]), lacune burden (AOR per additional lacune, 0.81 [95% CI, 0.67-0.98]), GCA score of 2 to 3 compared with 0 (AOR, 0.47 [95% CI, 0.25-0.87]), and total brain frailty score of 3 compared with 0 (AOR, 0.33 [95% CI, 0.16-0.70]) each remained associated with lower odds of mRS score of 0 to 1. Total Fazekas score remained significant after Hochberg correction (Figure 1, Figure 2, and eTable 7 in Supplement 1). On adjusted sensitivity analyses with MRI-evaluated markers, in addition to total Fazekas score, lacune burden was also associated with lower odds of mRS score of 0 to 1 (AOR per additional lacune, 0.70 [95% CI, 0.53-0.92]) but was not significant after Hochberg correction (Figure 3, Figure 4, and eTable 8 in Supplement 1). For additional MRI-specific markers (EPVS, CMB, CSS, and SVD score), total EPVS score (AOR per EPVS point increase, 0.85 [95% CI, 0.73-0.99]) and total SVD score of 3 to 4 compared with 0 (AOR, 0.28 [95% CI, 0.10-0.76]) were both associated with lower odds of mRS scores of 0 to 1 on adjusted analyses but were not significant after Hochberg correction (Figure 3, Figure 4, and eTable 8 in Supplement 1). All adjusted models had satisfactory calibration as indicated by fair to good AUROCs (eTables 9 and 10 in Supplement 1). An additional sensitivity analysis was performed to additionally adjust for patients who underwent treatment with EVT (502 [32.0%]), as well as those with hypertension (715 [49.1%]) or diabetes (274 [18.8%]). Following EVT, 33 patients (6.6%) had a modified treatment in cerebral infarction score of 0; 3 (0.6%), 1; 22 (4.4%), 2a; 172 (34.2%), 2b; 41 (8.2%), 2c; and 231 (46.0%), 3. On this sensitivity analysis, total brain frailty score remained negatively associated with 90-day mRS score of 0 to 1 (AOR, 0.68 [95% CI, 0.59-0.79]).

For ordinal mRS, total Fazekas score, presence of lacunes or chronic infarctions, GCA score, and total brain frailty score were associated with worse 90-day mRS on adjusted analyses (adjusted common OR [ACOR] for total brain frailty score of 2 vs 0, 2.24 [95% CI, 1.43-3.51]; ACOR for score 3 vs 0, 3.15 [95% CI, 1.87-5.33]; ACOR for total Fazekas score, 2.80 [95% CI, 1.88-4.16]; ACOR for GCA score, 2.65 [95% CI, 1.63-4.32]); after Hochberg correction, total Fazekas score, GCA score, and total brain frailty score each remained significant (Figure 1 and eFigure 2 and eTable 11 in Supplement 1). On adjusted sensitivity analyses with MRI markers, lacune burden and total brain frailty score were also associated with worse ordinal mRS scores (ACOR per additional lacune, 1.17 [95% CI, 1.03-1.32]; ACOR for total brain frailty score 3 vs 0, 3.31 [95% CI, 1.38-7.98]) but were not significant after Hochberg correction (Figure 3 and eFigure 3 and eTable 12 in Supplement 1). No MRI-specific markers were associated with ordinal mRS score on adjusted analyses.

For safety outcomes, the presence of chronic infarctions was positively associated with symptomatic ICH (AOR, 2.73 [95% CI, 1.13-6.59]) (eTable 13 in Supplement 1). No markers were associated with ICH visible on neuroimaging (eTable 14 in Supplement 1) or ICH severity on NCCT (eTable 15 in Supplement 1) on adjusted analyses. Total Fazekas score (AOR for total Fazekas score 3-6 vs 0, 1.75 [95% CI, 1.02-3.00]), GCA score (AOR for GCA score 2-3 vs 0, 2.88 [95% CI, 1.51-5.49]), and total brain frailty score (AOR for brain frailty score 3 vs 0, 2.17 [95% CI, 1.09-4.33]) were positively associated with mortality (eTable 16 in Supplement 1). On adjusted sensitivity analyses with MRI markers, CMB burden (AOR per additional CMB, 1.20 [95% CI, 1.01-1.43]), presence of CSS (AOR, 14.83 [95% CI, 1.19-184.37]), CSS burden (AOR per additional affected sulcus, 3.30 [95% CI, 1.24-8.77]), and total SVD score (AOR of symptomatic ICH for total SVD score 2 vs 0, 18.91 [95% CI, 1.25-286.93]) were each positively associated with symptomatic ICH (eTable 17 in Supplement 1). Higher total Fazekas score (AOR for ICH visible on neuroimaging for total Fazekas score 1-2 vs 0, 0.47 [95% CI, 0.22-0.98]) and total EPVS score (AOR for ICH visible on neuroimaging per EPVS point increase: 0.82 [95% CI, 0.69-0.97]) assessed on MRI were both negatively associated with ICH visible on neuroimaging (eTable 18 in Supplement 1). Higher CC:IT ratio (ACOR, 0.92 [95% CI, 0.84-0.99]) on MRI was associated with decreased ICH severity, while CMB burden (ACOR per additional CMB, 1.09 [95% CI, 1.02-1.16]) was associated with increased ICH severity (eTable 19 in Supplement 1). No MRI marker was associated with mortality in adjusted analyses (eTable 20 in Supplement 1). None of the markers assessed on NCCT or MRI were associated with any safety outcome in adjusted analyses after Hochberg correction.

There was an interaction by thrombolytic type for 2 brain frailty markers examined, subcortical atrophy (CC:IT ratio) and CSS. Patients with CC:IT ratios in the third or fourth quartile on NCCT who received tenecteplase had lower odds of achieving mRS scores of 0 to 1 and had higher ordinal mRS scores than those who received alteplase (AOR for mRS 0-1 with tenecteplase vs alteplase in patients with CC:IT Q3 0.40 [95% CI, 0.21-0.76]; AOR of patients with CC:IT in quartile 4, 0.44 [95% CI, 0.23-0.83]); results were similar when examining MRI-based CC:IT ratio. In patients who received tenecteplase, higher CC:IT ratios were also positively associated with ICH visible on both NCCT and MRI (AOR for ICH visible on neuroimaging with tenecteplase vs alteplase in patients with higher CC:IT ratio on NCCT, 1.07 [95% CI, 1.01-1.13]; AOR for MRI, 1.10 [95% CI, 1.01-1.21]), as well as ICH severity on MRI (ACOR for ICH severity with tenecteplase vs alteplase in patients with higher CC:IT ratio:, 1.11 [95% CI, 1.02-1.21]). The presence and burden of CSS were positively associated with ICH severity in patients treated with tenecteplase compared with alteplase (ACOR of ICH severity with tenecteplase vs alteplase in patients with for CSS presence, 10.11 [95% CI, 1.06-96.3]; ACOR per additional CSS lesion, 4.74 [95% CI, 1.81-12.4]).

## Discussion

In this post hoc cohort analysis of the AcT trial, increasing brain frailty was associated with lower odds of excellent functional outcomes and worse overall functional outcomes at 90 days in patients with AIS treated with intravenous thrombolysis, across various neuroimaging measures of brain frailty and independently of known prognostic factors. Specifically, following correction for multiple comparisons, the burden of WMC (total Fazekas score) was associated with lower odds of achieving excellent functional outcome. The burden of WMC and GCA score, as well as a total brain frailty score capturing the aggregate burden of WMC, cortical or subcortical atrophy, and presence of lacunes or chronic infarctions, were independently associated with worse ordinal functional outcome. No imaging markers remained associated with examined safety outcomes (symptomatic ICH, ICH visible on neuroimaging, severity of ICH, or mortality) following correction for multiple comparisons.

The brain frailty score was adopted from a prior analysis of the Efficacy of Nitric Oxide in Stroke (ENOS) trial of patients with ischemic or hemorrhagic stroke, in which brain frailty was similarly associated with worse 90-day functional outcome.^2^ Recent analyses of patients from the Safety and Efficacy of Nerinetide in Subjects Undergoing Endovascular Thrombectomy for Stroke (ESCAPE-NA1) clinical trial found that neuroimaging markers of brain frailty mediated most of the association of age with 90-day mRS score.^17^ Assessment of MRI scans from the Stroke Imaging Repository/Virtual International Stroke Trials Archive (VISTA) Imaging consortium showed that severe WMC and total SVD score were associated with functional disability in 259 patients with AIS receiving thrombolytic treatment with alteplase.^7^ Our results add to this literature by analyzing markers of brain frailty (both atrophy and SVD) on both NCCT and MRI in a large sample from a pragmatic randomized clinical trial that included patients with AIS treated with either alteplase or tenecteplase.

Concerns have been raised regarding the use of reperfusion therapies in patients with underlying brain frailty markers, particularly a high burden of WMC or microbleeds, due to the risk of postthrombolysis symptomatic ICH suggested by prior observational studies, including a meta-analysis of 15 observational studies.^18,19^ Considerations about the underlying burden of WMC and microbleeds may also dissuade physicians from administering thrombolysis in patients with prestroke disability and dementia.^20^ However, our study found no consistent association between brain frailty burden and ICH-related safety outcomes (symptomatic ICH, ICH visible on neuroimaging, or ICH severity), despite the association with poorer functional outcome, consistent with a recent meta-analysis of 30 studies showing that WMC, atrophy, and SVD burden were negatively associated with functional outcome but not with radiographic or symptomatic ICH.^21^ We did find associations between CMB and CSS burden and both symptomatic ICH and ICH severity, and although these associations were no longer present after adjustment for multiple comparisons, incorporating these markers in brain frailty analyses may enhance hemorrhagic risk stratification. In 459 patients with AIS enrolled in the Efficacy and Safety of MRI-Based Thrombolysis in Wake-Up Stroke (WAKE-UP) trial, CMB presence was not associated with increased risk of symptomatic ICH in patients receiving alteplase vs placebo.^22^ These findings collectively suggest that underlying WMC and microbleeds should not by themselves preclude patients with AIS from receiving thrombolysis. Precisely how brain frailty contributes to poorer functional outcome after stroke without influencing rates of hemorrhage is likely multifactorial, involving structural and functional changes that affect poststroke recovery, including cerebral hypoperfusion,^23^ poor collateral circulation,^24^ and impaired structural connectivity.^25^ It is biologically plausible that the diminished resilience to injury attributed to brain frailty may be more relevant for longer-term processes such as neuroplasticity that contribute to functional recovery over several months and beyond, compared with shorter-term processes such as hemorrhagic transformation that typically manifest during 24 to 48 hours.^26^

When considering the overarching question of how to best apply these insights about brain frailty to the clinical setting, the tremendous variety of neuroimaging markers can seem burdensome for frontline clinicians. However, our findings highlight the burden of WMC and cortical atrophy as the brain frailty markers most robustly associated with postthrombolysis functional outcomes, which can simply be measured clinically using the Fazekas and GCA scores, respectively, but can also be assessed in combination with the presence of chronic infarcts and lacunes using the total brain frailty score. Clinical training and knowledge translation efforts regarding brain frailty in acute stroke could therefore focus on these key markers to maximize simplicity and efficiency. Given the feasibility and relevance of evaluating brain frailty on NCCT, our results suggest that brain frailty assessment on baseline NCCT may provide a more standardized measure of the underlying vulnerability that our field has hitherto sought to capture with premorbid disability measures such as the prestroke mRS, which was not designed for evaluation of prestroke functional assessment and has suboptimal validity.^27,28^ Brain frailty may therefore more accurately determine poststroke recovery and provide an alternative method for patient selection or stratification for clinical trials.

NCCT has been the primary imaging modality for acute stroke diagnosis and treatment selection due to its accessibility, speed, and affordability. For brain frailty assessment, MRI, when available, has the advantage over NCCT of detecting additional SVD markers (EPVS, CMB, and CSS). In our analyses, none of the individual MRI-specific markers, including the total SVD score obtained from MRI, were associated with functional outcome after correcting for multiple comparisons, unlike the NCCT-assessable markers such as WMC, GCA score, and total brain frailty score. Therefore, our results suggest that NCCT may be sufficient for assessing brain frailty in the acute stroke thrombolysis setting for informing prognostication. Whereas MRI-based brain frailty markers could only be assessed in a subset of our patients (n = 495), our MRI sample was nearly double that of the prior VISTA consortium study.

We observed an interaction with thrombolytic type for 2 markers. Notably, CC:IT ratio (subcortical atrophy) was associated with worse mRS outcomes, ICH visible on neuroimaging, and ICH severity in recipients of tenecteplase vs alteplase. It is possible that the increased fibrin specificity of tenecteplase and its higher rates of reperfusion may put patients with more frail brains at risk of reperfusion injury or hemorrhagic transformation.^29^ However, we did not observe interactions by thrombolytic type for symptomatic ICH or mortality, and there was no interaction for the association of total brain frailty with functional or safety outcomes by thrombolytic type, indicating that markers of brain frailty probably should not influence thrombolytic choice. These results are generally in line with those of prior trials^8,30^ demonstrating tenecteplase to be noninferior to alteplase for AIS.

### Limitations

This post hoc analysis has some limitations. While the trial-based data collection helped ensure high data completeness and quality for the core NCCT imaging and mRS outcomes and a large sample of participants from across Canada, the generalizability of our results to more diverse populations in everyday clinical practice may be limited. The 4.5-hour symptom onset window further limits the applicability of these results to patients arriving after this time frame. Given the pragmatic nature of the AcT trial, prestroke functional and cognitive assessments are unavailable for comparison with poststroke outcomes; therefore, residual confounding from patients with prestroke disability cannot be excluded. Additionally, follow-up MRI was performed at the discretion of each participating site, resulting in a reduced sample size of patients with available MRI scans for analysis of MRI-based brain frailty markers. Last, brain frailty is only a single aspect of multidimensional clinical frailty, and we lacked data from clinical frailty scales or measures such as sarcopenia to more deeply characterize frailty in our population.

## Conclusions

In this large cohort from the AcT trial, total brain frailty was associated with worse functional outcomes after thrombolysis for AIS. Incorporating markers of brain frailty—particularly WMC and cortical atrophy—into the assessment of AIS may help inform treatment expectations for patients who receive intravenous thrombolysis. Overall, our study emphasizes the value of considering brain frailty as part of neuroimaging assessments in stroke care and in clinical trials.

## Notes

Supplement 1. **eMethods.** Imaging Analyses **eFigure 1.** CONSORT (Consolidated Standards of Reporting Trials) Flow Diagram **eFigure 2.** Forest Plot of Adjusted Common Odds Ratios for the Secondary Outcome (Ordinal mRS) Stratified by Measures of Brain Frailty Assessed on NCCT **eFigure 3.** Forest Plot of Adjusted Common Odds ratios for the Secondary Outcome (Ordinal mRS) Stratified by Measures of Brain Frailty Assessed on MRI **eTable 1.** Scores Used for Analysis of Neuroimaging Markers **eTable 2.** Brant Tests for Ordinal mRS (Trichotomized) on NCCT **eTable 3.** Brant Tests for Ordinal mRS (Trichotomized) on MRI **eTable 4.** Baseline Characteristics of Participants Stratified by Total Brain Frailty Score Assessed on MRI **eTable 5.** Baseline Characteristics of Participants Stratified by Total Brain Frailty Score Assessed on MRI **eTable 6.** Interrater and Intrarater Reliability for Key Scales **eTable 7.** Association of Brain Frailty Markers Assessed on NCCT With Excellent Functional Outcome (mRS 0 to 1) **eTable 8.** Association of Brain Frailty Markers Assessed on MRI With Excellent Functional Outcome (mRS 0 to 1) **eTable 9.** AUROC for mRS 0 to 1 on NCCT **eTable 10.** AUROC for mRS 0 to 1 on MRI **eTable 11.** Association of Brain Frailty Markers Assessed on NCCT With Functional Outcome (Ordinal mRS) **eTable 12.** Association of Brain Frailty Markers Assessed on MRI With Functional Outcome (Ordinal mRS) **eTable 13.** Association of Brain Frailty Markers Assessed on NCCT With sICH **eTable 14.** Association of Brain Frailty Markers Assessed on NCCT With Radiographic ICH **eTable 15.** Association of Brain Frailty Markers Assessed on NCCT With ICH Severity (Ordinal Scale According to the Heidelberg Classification System) **eTable 16.** Association of Brain Frailty Markers Assessed on NCCT With Mortality (mRS 6) **eTable 17.** Association of Brain Frailty Markers Assessed on MRI With Symptomatic ICH **eTable 18.** Association of Brain Frailty Markers Assessed on MRI With Radiographic ICH **eTable 19.** Association of Brain Frailty Markers Assessed on MRI With ICH Severity **eTable 20.** Association of Brain Frailty Markers Assessed on MRI With Mortality (mRS 6)
eReferences

Supplement 2.
Data Sharing Statement
