# Supplementary material for: Brain Frailty and Functional Outcomes After Thrombolysis for Acute Ischemic Stroke
Source: JAMA Netw Open. 2025 Sep 30;8(9):e2534941. doi: 10.1001/jamanetworkopen.2025.34941 (PMC12485648; doi:10.1001/jamanetworkopen.2025.34941)
Supplement: Supplement 1. — eMethods. Imaging Analyses eFigure 1. CONSORT (Consolidated Standards of Reporting Trials) Flow Diagram eFigure 2. Forest Plot of Adjusted Common Odds Ratios for the Secondary Outcome (Ordinal mRS) Stratified by Measures of Brain Frailty Assessed on NCCT eFigure 3. Forest Plot of Adjusted Common Odds ratios for the Secondary Outcome (Ordinal mRS) Stratified by Measures of Brain Frailty Assessed on MRI eTable 1. Scores Used for Analysis of Neuroimaging Markers eTable 2. Brant Tests for Ordinal mRS (Trichotomized) on NCCT eTable 3. Brant Tests for Ordinal mRS (Trichotomized) on MRI eTable 4. Baseline Characteristics of Participants Stratified by Total Brain Frailty Score Assessed on MRI eTable 5. Baseline Characteristics of Participants Stratified by Total Brain Frailty Score Assessed on MRI eTable 6. Interrater and Intrarater Reliability for Key Scales eTable 7. Association of Brain Frailty Markers Assessed on NCCT With Excellent Functional Outcome (mRS 0 to 1) eTable 8. Association of Brain Frailty Markers Assessed on MRI With Excellent Functional Outcome (mRS 0 to 1) eTable 9. AUROC for mRS 0 to 1 on NCCT eTable 10. AUROC for mRS 0 to 1 on MRI eTable 11. Association of Brain Frailty Markers Assessed on NCCT With Functional Outcome (Ordinal mRS) eTable 12. Association of Brain Frailty Markers Assessed on MRI With Functional Outcome (Ordinal mRS) eTable 13. Association of Brain Frailty Markers Assessed on NCCT With sICH eTable 14. Association of Brain Frailty Markers Assessed on NCCT With Radiographic ICH eTable 15. Association of Brain Frailty Markers Assessed on NCCT With ICH Severity (Ordinal Scale According to the Heidelberg Classification System) eTable 16. Association of Brain Frailty Markers Assessed on NCCT With Mortality (mRS 6) eTable 17. Association of Brain Frailty Markers Assessed on MRI With Symptomatic ICH eTable 18. Association of Brain Frailty Markers Assessed on MRI With Radiographic ICH eTable 19. Association of Brain Frailty Markers Assessed on MR [file jamanetwopen-e2534941-s001.pdf]

Supplementary Online Content

Loewen SP, Singh N, Alhabli I, et al. Brain frailty and functional outcomes after thrombolysis for acute ischemic stroke. *JAMA Netw Open.* 2025;8(10):e2534941. doi:10.1001/jamanetworkopen.2025.34941

eMethods. Imaging Analyses

eFigure 1. CONSORT (Consolidated Standards of Reporting Trials) Flow Diagram

eFigure 2. Forest Plot of Adjusted Common Odds Ratios for the Secondary Outcome (Ordinal mRS) Stratified by Measures of Brain Frailty Assessed on NCCT

eFigure 3. Forest Plot of Adjusted Common Odds ratios for the Secondary Outcome (Ordinal mRS) Stratified by Measures of Brain Frailty Assessed on MRI

eTable 1. Scores Used for Analysis of Neuroimaging Markers

eTable 2. Brant Tests for Ordinal mRS (Trichotomized) on NCCT

eTable 3. Brant Tests for Ordinal mRS (Trichotomized) on MRI

eTable 4. Baseline Characteristics of Participants Stratified by Total Brain Frailty Score Assessed on MRI

eTable 5. Baseline Characteristics of Participants Stratified by Total Brain Frailty Score Assessed on MRI

eTable 6. Interrater and Intrarater Reliability for Key Scales

eTable 7. Association of Brain Frailty Markers Assessed on NCCT With Excellent Functional Outcome (mRS 0 to 1)

eTable 8. Association of Brain Frailty Markers Assessed on MRI With Excellent Functional Outcome (mRS 0 to 1)

eTable 9. AUROC for mRS 0 to 1 on NCCT

eTable 10. AUROC for mRS 0 to 1 on MRI

eTable 11. Association of Brain Frailty Markers Assessed on NCCT With Functional Outcome (Ordinal mRS)

eTable 12. Association of Brain Frailty Markers Assessed on MRI With Functional Outcome (Ordinal mRS)

© 2025 Loewen SP et al. *JAMA Network Open*.

eTable 13. Association of Brain Frailty Markers Assessed on NCCT With sICH

eTable 14. Association of Brain Frailty Markers Assessed on NCCT With Radiographic ICH

eTable 15. Association of Brain Frailty Markers Assessed on NCCT With ICH Severity (Ordinal Scale According to the Heidelberg Classification System)

eTable 16. Association of Brain Frailty Markers Assessed on NCCT With Mortality (mRS 6)

eTable 17. Association of Brain Frailty Markers Assessed on MRI With Symptomatic ICH

eTable 18. Association of Brain Frailty Markers Assessed on MRI With Radiographic ICH

eTable 19. Association of Brain Frailty Markers Assessed on MRI With ICH Severity

eTable 20. Association of Brain Frailty Markers Assessed on MRI With Mortality (mRS 6)

eReferences

This supplementary material has been provided by the authors to give readers additional information about their work.

eMethods. Imaging Analyses

Cortical atrophy was assessed using the global cortical atrophy (GCA) scale, a pragmatic, qualitative scale that rates the degree of atrophy from 0 (no atrophy) to 3 (severe atrophy) based on the width of sulci and volume of gyri.[1] For subcortical atrophy, the hemi-intercaudate distance (h-ICD) was measured on the contralateral side from the ischemic event and multiplied by 2 to obtain the ICD, which accounts for potential complications such as edema at the infarct site.[2] The ratio of the ICD to the inner-table-width was then calculated (CC/IT ratio) and used to evaluate subcortical atrophy. Chronic cerebral infarctions were characterized as any cortical or subcortical tissue loss exceeding an axial diameter of 15 mm. Chronic infarction burden was characterized based on the total number of infarcts present. Lacunes were defined as subcortical infarcts ranging from 3-15 mm.[3] The presence of chronic infarcts and/or lacunes were combined and characterized as vascular lesions. The degree of white matter change (WMC; also known as leukoaraiosis, visible as hypodensity on NCCT and hyperintensity on MRI) was assessed separately in periventricular and deep locations using the Fazekas scale,[4] with each location graded from 0-3, as has been done previously using both NCCT and MRI scans.[5,6] We have previously compared our team's NCCT and MRI ratings of these aforementioned brain frailty measures in both AcT and other datasets, and found them to have excellent agreement (Gwet's agreement coefficient >0.78).[7]

Follow-up MRI scans were performed within 24-72 hours following initial presentation and were further assessed for cerebral microbleeds (CMB), enlarged perivascular spaces (EPVS), and cortical superficial siderosis (CSS), features not visible on NCCT. Microbleeds were defined as small (<10 mm), round or ovoid hypointense foci on SWI, GRE, or T2* sequences, and assessed using the Brain Observer Micro-Bleed Scale.[8] EPVS were defined as small punctate or linear hyperintensities in the basal ganglia (BG) or centrum semiovale (CS) on T2 or FLAIR sequences and rated on an ordinal 5-point scale (0 [none], 1 [mild, 1-10], 2 [moderate, 11-20], 3 [frequent, 21-40], and 4 [severe, >40]).[9] EPVS was also assessed in the midbrain and characterized here simply as being present or absent as in the originally proposed scale.[9] The presence of superficial siderosis was assessed on SWI, GRE, or T2* sequences in the frontal, parietal, temporal, occipital, insular, and cerebellar regions. CSS burden was characterized based on the number of affected sulci. FLAIR sequences were used to detect chronic infarcts and lacunes on MRI.

For analyses of neuroimaging markers, we combined the individual scores for periventricular and deep WMC into a total Fazekas score and separated these total scores into three categories (0, 1-2, 3-6). GCA scores were also separated into three categories (0, 1, 2-3). These categories for Fazekas scores and GCA scores were chosen based on the distribution of the data across our population. For subcortical atrophy, CC/IT ratios were divided into quartiles (Q1:<0.10; Q2:0.10-0.12; Q3:0.12-0.15; and Q4:>0.15). A total EPVS score (0–9) was calculated by summing the individual BG, CS, and midbrain EPVS scores. A total SVD score based on a previously validated composite score[10] was calculated from MRI-based markers whereby 1 point each was added for severe WMC (total Fazekas score 3-6), moderate to severe EPVS in BG (EPVS score 2-4), any lacunes, and any CMB (maximum 4 of 4). Total brain frailty scores were calculated for both CT-based markers and MRI-based markers. These scores were based on prior work by investigators of the Efficacy of Nitric Oxide in Stroke (ENOS) trial and have previously been used to assess brain frailty in acute stroke patients.[11] Both scores were ordinal 4-point scales whereby 1 point each was added for severe WMC (total Fazekas score 3-6), severe cortical (GCA score 2-3) or subcortical atrophy (highest CC/IT ratio quartile), and any vascular lesions (maximum 3 of 3).[11] No additional weighting was applied to any of the composite scores.

© 2025 Loewen SP et al. *JAMA Network Open*.

eFigure 1. CONSORT (Consolidated Standards of Reporting Trials) Flow Diagram

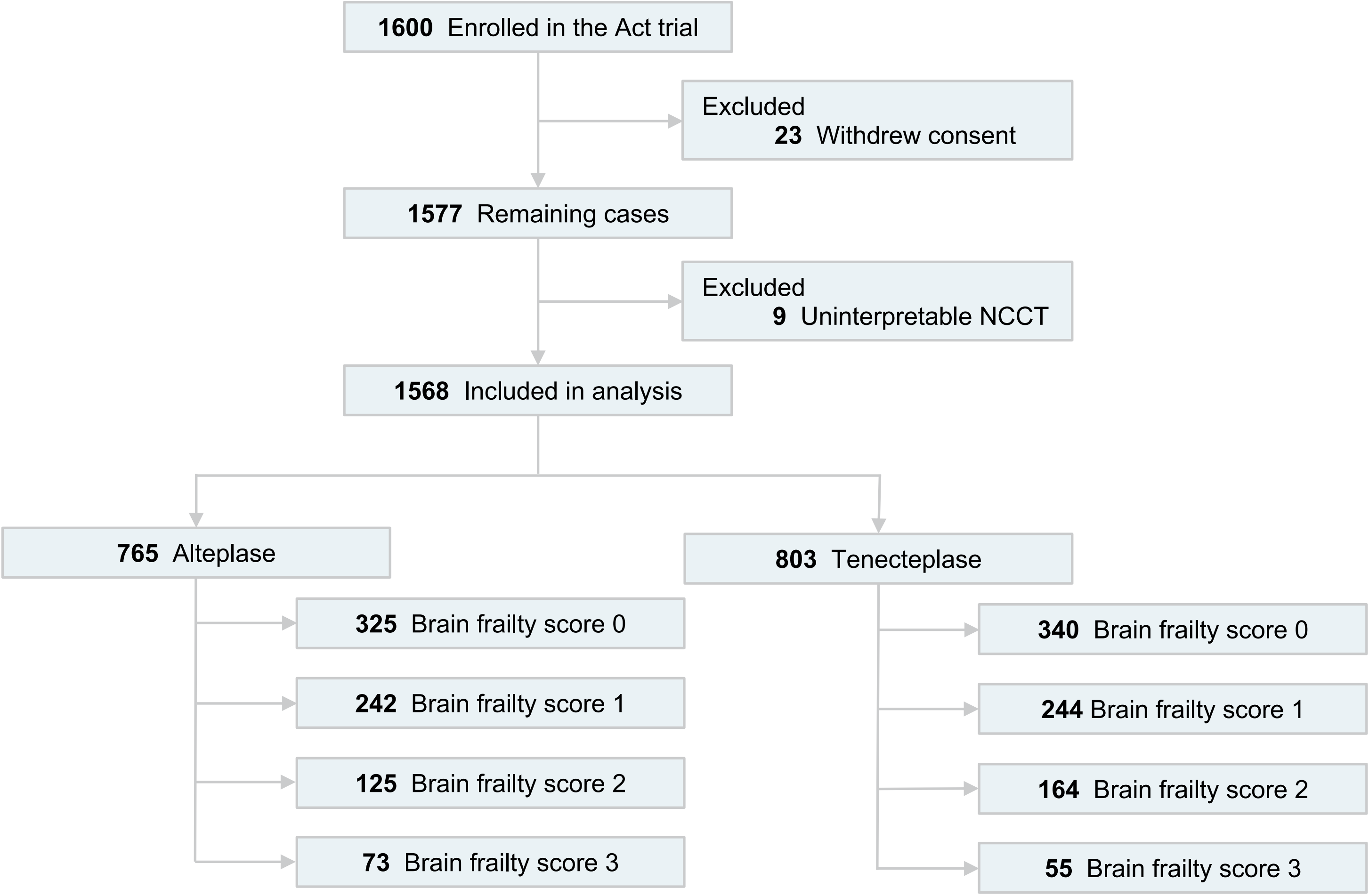

Abbreviations: NCCT = non-contrast computed tomography.

© 2025 Loewen SP et al. *JAMA Network Open*.

eFigure 2. Forest Plot of Adjusted Common Odds Ratios for the Secondary Outcome (Ordinal mRS) Stratified by Measures of Brain Frailty Assessed on NCCT

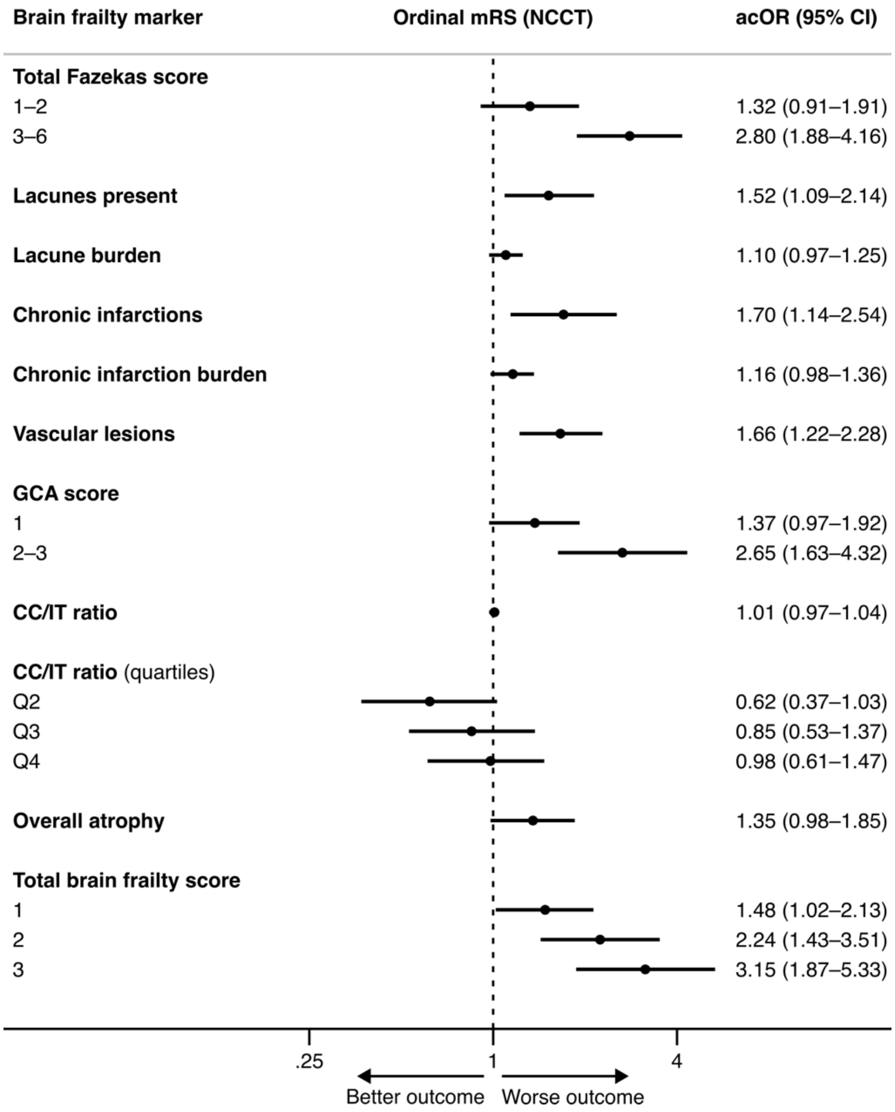

Adjustments were made for age, sex, pretreatment NIHSS, and stroke symptom onset-to-needle time as fixed-effects variables and participating site as a random-effects variable.
Abbreviations: CC/IT = intercaudate distance to inner-table-width ratio; GCA = global cortical atrophy; mRS = modified Rankin scale; NCCT = non-contrast computed tomography.

© 2025 Loewen SP et al. *JAMA Network Open*.

eFigure 3. Forest Plot of Adjusted Common Odds ratios for the Secondary Outcome (Ordinal mRS) Stratified by Measures of Brain Frailty Assessed on MRI

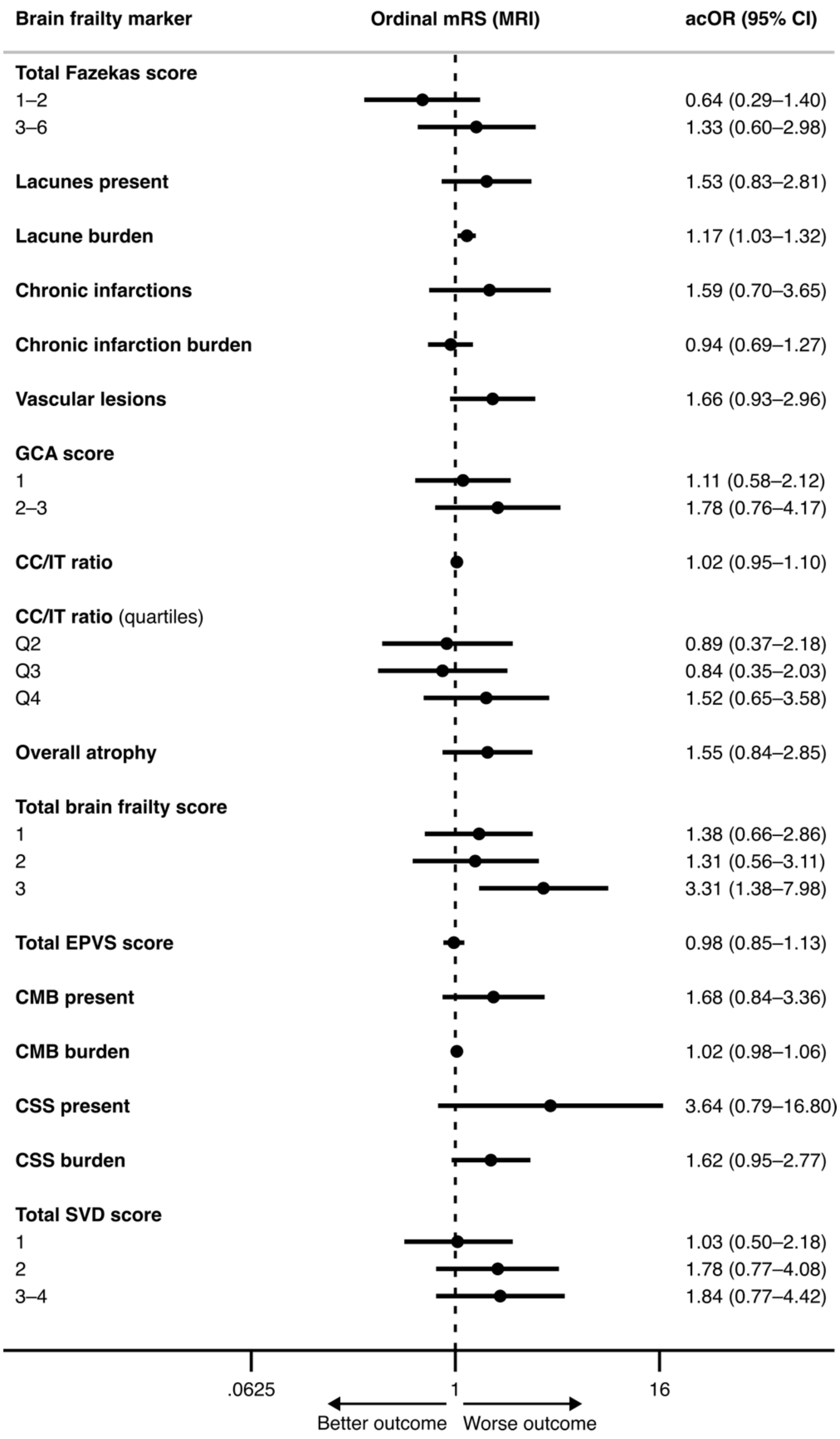

© 2025 Loewen SP et al. *JAMA Network Open*.

Adjustments were made for age, sex, pretreatment NIHSS, and stroke symptom onset-to-needle time as fixed-effects variables and participating site as a random-effects variable.
Abbreviations: CC/IT = intercaudate distance to inner-table-width ratio; CMB = cerebral microbleeds; CSS = cortical superficial siderosis; SVD = small vessel disease; EPVS = enlarged perivascular spaces; GCA = global cortical atrophy; MRI = magnetic resonance imaging; mRS = modified Rankin scale.

© 2025 Loewen SP et al. *JAMA Network Open*.

eTable 1. Scores Used for Analysis of Neuroimaging Markers

Score	Marker/type	Definition	Range/scale	Imaging modality
Fazekas scale	White matter change/ leukoaraiosis	White matter change in periventricular and deep white matter	0 (absent), 1 (caps or pencil-thin lining; punctate foci), 2 (smooth halo; beginning confluence), 3 (irregular periventricular signal extending into deep white matter; large confluent areas)	NCCT, MRI
Total Fazekas score	White matter change/ leukoaraiosis	Sum of individual periventricular and deep white matter Fazekas scores	0 to 6 (0-3 from periventricular white matter and 0-3 from deep white matter)	NCCT, MRI
GCA score	Cortical atrophy	Global cortical atrophy based on width of sulci and volume of gyri	0 (no atrophy), 1 (mild), 2 (moderate), 3 (severe)	NCCT, MRI
CC/IT ratio	Subcortical atrophy	Ratio of intercaudate distance to inner-table-width	Quartiles: Q1: <0.10, Q2: 0.10-0.12, Q3: 0.12-0.15, Q4: >0.15	NCCT, MRI
EPVS scale	Enlarged perivascular spaces	EPVS assessed in BG and CS	0 (none), 1 (mild, 1-10), 2 (moderate, 11-20), 3 (frequent, 21-40), and 4 (severe, >40)	MRI
Total EPVS score	Enlarged perivascular spaces	Sum of individual BG, CS, and midbrain EPVS scores	0 to 9 (0-4 from BG, 0-4 from CS, 0-1 from midbrain)	MRI
Total SVD score	Composite small vessel disease	Composite score combining WMC, EPVS, lacunes, CMB	0 to 4 (1 point each for severe WMC (total Fazekas score 3-6), moderate to severe EPVS in BG (EPVS score 2-4), any lacunes, any CMB	MRI
Total brain frailty score	Composite brain frailty	Composite score combining WMC, cortical atrophy, subcortical atrophy, vascular lesions (chronic infarcts and/or lacunes)	0 to 3 (1 point each for severe WMC (total Fazekas score 3-6), severe cortical (GCA score 2-3) or subcortical atrophy (highest CC/IT ratio quartile), any vascular lesions	NCCT, MRI

Abbreviations: BG = basal ganglia; CC/IT = intercaudate distance to inner-table-width ratio; CS = centrum semiovale; EPVS = enlarged perivascular spaces; GCA = global cortical atrophy; MRI = magnetic resonance imaging; NCCT = non-contrast computed tomography; SVD = small vessel disease; WMC = white matter change.

© 2025 Loewen SP et al. *JAMA Network Open*.

eTable 2. Brant Tests for Ordinal mRS (Trichotomized) on NCCT

Variables significant at *p*<0.05 are bolded.

NCCT Brain Frailty Marker	Chi-squared	*p* value	Adjusted chi-squared	*p* value
Total Fazekas score	1.60	0.21	4.53	010
Lacunes present	0.26	0.61	1.53	0.98
Lacune burden	2.07	0.15	2.88	0.90
≥1 Chronic infarction	3.68	0.06	3.96	0.78
Chronic infarction burden	3.47	0.06	3.57	0.83
Any lacune or chronic infarct	1.65	0.98	0.12	0.73
GCA score	0.01	0.92	1.51	0.99
Subcortical atrophy (CC/IT ratio)	5.74	**0.02**	2.86	0.90
Subcortical atrophy (CC/IT ratio quartile)	2.21	0.14	4.63	0.95
Severe cortical or subcortical atrophy	1.48	0.22	2.47	0.93
Brain frailty score	1.69	0.19	4.14	0.97

Abbreviations: CC/IT = intercaudate distance to inner-table-width ratio; GCA = global cortical atrophy; mRS = modified Rankin scale; NCCT = non-contrast computed tomography.

© 2025 Loewen SP et al. *JAMA Network Open*.

eTable 3. Brant Tests for Ordinal mRS (Trichotomized) on MRI

Variables significant at *p*<0.05 are bolded.

MRI Brain Frailty Marker	Chi-squared	*p* value	Adjusted chi-squared	*p* value
Total Fazekas score	1.13	0.29	6.07	0.73
Lacunes present	0.02	0.89	2.33	0.94
Lacune burden	1.22	0.27	4.50	0.72
≥1 Chronic infarction	0.11	0.75	3.47	0.84
Chronic infarction burden	0.37	0.54	7.00	0.43
Any lacune or chronic infarct	0.27	0.61	2.65	0.92
GCA score	0.52	0.47	5.75	0.77
Subcortical atrophy (CC/IT ratio)	1.42	0.23	3.02	0.88
Subcortical atrophy (CC/IT ratio quartiles)	0.19	0.66	NA	NA
Severe cortical or subcortical atrophy	0.02	0.88	2.36	0.94
Brain frailty score	0.27	0.60	5.21	0.92
Total EPVS score	2.65	0.10	6.86	0.44
≥1 CMB	4.56	**0.03**	9.50	0.22
CMB burden	1.53	0.22	3.63	0.82
CSS present	1.06	0.30	3.69	0.82
CSS burden (total number of affected sulci)	1.44	0.23	3.00	0.89
SVD score	2.58	0.11	9.05	0.62

Abbreviations: CC/IT = intercaudate distance to inner-table-width ratio; CMB = cerebral microbleeds; CSS = cortical superficial siderosis; EPVS = enlarged perivascular spaces; GCA = global cortical atrophy; MRI = magnetic resonance imaging; mRS = modified Rankin scale; SVD = small vessel disease.

© 2025 Loewen SP et al. *JAMA Network Open*.

eTable 4. Baseline Characteristics of Participants Stratified by Total Brain Frailty Score Assessed on MRI

Characteristic	Brain frailty Score 0 (n = 206)	Brain frailty Score 1 (n = 134)	Brain frailty Score 2 (n = 96)	Brain frailty Score 3 (n = 59)	*p* value
Age, y (IQR)	61 (51-70)	71 (63-79)	79 (72-86)	81 (75-86)	**<0.001**
Sex					
Female	98 (47.6)	60 (44.8)	43 (44.8)	33 (55.9)	0.50
Male	108 (52.4)	74 (55.2)	53 (55.2)	26 (44.1)	
Baseline NIHSS score, median (IQR)	8 (5-13)	8 (5-15)	8 (5-14)	7 (6-13)	0.90
Baseline ASPECTS, median (IQR)	9 (8-10)	9 (8-10)	9 (9-10)	9 (8-10)	**0.03**
Stroke symptom onset to needle time (minutes, intravenous thrombolysis start)	37 (29-53)	37 (27-49)	36.5 (30-49.5)	37 (29-48.5)	0.85
Comorbidities (n = 1456)					
Hypertension	86 (45.7)	52 (42.3)	61 (68.5)	27 (50.9)	**0.001**
Diabetes	31 (16.5)	26 (21.1)	19 (21.4)	14 (26.4)	0.39
Atrial fibrillation	18 (9.6)	17 (13.8)	18 (20.2)	8 (15.1)	0.11
Dyslipidemia	16 (8.5)	11 (8.9)	6 (6.7)	4 (7.6)	0.94
Coronary artery disease	2 (1.1)	3 (2.4)	5 (5.6)	4 (7.6)	**0.04**
Smoking (current/past)	4 (2.1)	2 (1.6)	1 (1.2)	2 (3.8)	0.73
Previous stroke	1 (0.5)	0 (0.0)	0 (0.0)	0 (0.0)	0.70
Thrombolytic treatment					
Tenecteplase	109 (52.9)	68 (50.8)	61 (63.5)	27 (45.8)	0.13
Alteplase	97 (47.1)	66 (49.2)	35 (36.5)	32 (54.2)	
Total Fazekas Score					
0	96 (48.0)	21 (15.9)	2 (2.1)	0 (0.0)	**<0.001**
1-2	104 (52.0)	72 (54.6)	25 (26.0)	0 (0.0)	
3-6	0 (0.0)	39 (29.6)	69 (71.9)	59 (100.0)	
Lacunes present	0 (0.0)	27 (20.5)	47 (49.0)	50 (84.8)	**<0.001**
Lacune burden, median (IQR)	0 (0-0)	0 (0-0)	0 (0-2)	2 (1-4)	**<0.001**
≥1 Chronic infarction	5 (2.4)	13 (9.9)	21 (22.3)	18 (30.5)	**<0.001**
Chronic infarction burden, median (IQR)	0 (0-0)	0 (0-0)	0 (0-0)	0 (0-1)	**<0.001**
Any lacune or chronic infarct	0 (0.0)	44 (33.3)	60 (62.5)	59 (100.0)	**<0.001**
Global cortical atrophy score					
0	140 (70.4)	56 (42.7)	31 (32.3)	2 (3.4)	**<0.001**
1	59 (29.6)	63 (48.1)	39 (40.6)	26 (44.1)	
2-3	0 (0.0)	12 (9.2)	26 (27.1)	31 (52.5)	
CC/IT ratio, median (IQR)	0.11 (0.09-0.13)	0.13 (0.11-0.16)	0.16 (0.13-0.19)	0.17 (0.15-0.19)	**<0.001**
Severe cortical or subcortical atrophy	0 (0.0)	51 (38.1)	63 (65.6)	59 (100.0)	**<0.001**
Total EPVS score					
0	99 (51.0)	50 (39.1)	18 (19.3)	4 (7.0)	**<0.001**
1-3	72 (37.1)	60 (46.9)	49 (52.7)	32 (56.1)	

© 2025 Loewen SP et al. *JAMA Network Open*.

4-6	21 (10.8)	15 (11.7)	20 (21.5)	15 (26.3)	
7-9	2 (1.0)	3 (2.3)	6 (6.5)	6 (10.5)	
≥1 CMB	18 (9.2)	22 (17.2)	28 (30.4)	21 (36.2)	**<0.001**
CMB burden, median (IQR)	0 (0-0)	0 (0-0)	0 (0-1)	0 (0-1)	**<0.001**
CSS present	1 (0.5)	7 (5.5)	3 (3.3)	4 (7.0)	**0.03**
CSS burden (total number of affected sulci), median (IQR)	0 (0-0)	0 (0-0)	0 (0-0)	0 (0-0)	**0.03**
SVD score					
0	181 (87.9)	57 (42.5)	6 6.3)	0 (0.0)	**<0.001**
1	24 (11.6)	54 (40.3)	26 (27.1)	5 8.5)	
2	1 (0.5)	19 (14.2)	43 (44.8)	19 (32.2)	
3-4	0 (0.0)	4 (3.0)	21 (21.9)	35 (59.3)	

Abbreviations: ASPECTS = Alberta Stroke Program Early CT Score; CC/IT = intercaudate distance to inner-table-width ratio; CMB = cerebral microbleeds; CSS = cortical superficial siderosis; EPVS = enlarged perivascular spaces; IQR = interquartile range; MRI = magnetic resonance imaging; NIHSS = National Institutes of Health stroke scale; SVD = small vessel disease.

© 2025 Loewen SP et al. *JAMA Network Open*.

eTable 5. Baseline Characteristics of Participants Stratified by Total Brain Frailty Score Assessed on MRI

Characteristic	**No MRI (n = 1073)**	**MRI (n = 495)**	***p* value**
Age, y (IQR)	75 (65-84)	70 (58-81)	**<0.001**
Sex			
Female	517 (48.2)	234 (47.3)	0.74
Male	556 (51.8)	261 (52.7)	
Baseline NIHSS score, median (IQR)	11 (6-17)	8 (5-13)	**<0.001**
Baseline ASPECTS, median (IQR)	9 (8-10)	9 (8-10)	0.86
Stroke symptom onset to needle time (minutes, intravenous thrombolysis start)	36 (27-49)	37 (29-50)	0.10
Comorbidities (n = 1456)			
Hypertension	492 (49.2)	226 (49.9)	0.81
Diabetes	186 (18.6)	90 (19.9)	0.57
Atrial fibrillation	130 (13.0)	61 (13.5)	0.81
Dyslipidemia	39 (3.9)	37 (8.2)	**0.001**
Coronary artery disease	13 (1.3)	14 (3.1)	**0.02**
Smoker (current/past)	8 (0.8)	9 (2.0)	0.05
Previous stroke	11 (1.1)	1 (0.22)	0.09
Thrombolytic treatment			
Tenecteplase	538 (50.1)	265 (53.5)	0.21
Alteplase	535 (49.9)	230 (46.5)	
Endovascular thrombectomy	358 (33.2)	147 (29.7)	0.16
Total Fazekas Score			
0	552 (51.3)	238 (48.5)	0.15
1-2	293 (27.2)	157 (31.0)	
3-6	232 (21.5)	96 (19.5)	
Lacunes present	219 (20.3)	150 (30.6)	**<0.001**
Lacune burden, median (IQR)	0 (0-0)	0 (0-1)	**0.001**
≥1 Chronic infarction	167 (15.5)	57 (11.6)	**0.04**
Chronic infarction burden, median (IQR)	0 (0-0)	0 (0-0)	0.83
Any lacune or chronic infarct	340 (31.6)	180 (36.7)	**0.047**
Global cortical atrophy score			
0	539 (50.0)	212 (43.2)	**<0.001**
1	392 (36.4)	233 (47.4)	
2-3	146 (13.6)	46 (9.4)	
CC/IT ratio, median (IQR)	0.13 (0.11-0.16)	0.13 (0.10-0.15)	**0.03**
Severe cortical or subcortical atrophy	442 (41.0)	158 (32.2)	**0.001**

Abbreviations: ASPECTS = Alberta Stroke Program Early CT Score; CC/IT = intercaudate distance to inner-table-width ratio; GCA = global cortical atrophy; IQR = interquartile range; MRI = magnetic resonance imaging; NIHSS = National Institutes of Health stroke scale.

© 2025 Loewen SP et al. *JAMA Network Open*.

eTable 6. Interrater and Intrarater Reliability for Key Scales

Variable	Inter-rater agreement		Intra-rater
	% Agreement*	Gwet's AC1	Gwet's AC1
**Fazekas score**	0.84‡	0.64†	0.69†
**GCA**	0.85‡	0.73†	0.61†
**CC/IT Ratio**	0.97‡	0.96‡	0.87‡
**Lacunes**	0.75†	0.63†	0.92‡
**Chronic infarcts**	0.91‡	0.95‡	1.00‡

*For binary classification as none-mild vs. moderate-severe (for atrophy, Fazekas) or as present vs. absent (for lacunes and chronic infarcts); for CC/IT ratio, it is for classification by quartile.

† = substantial agreement (0.61-0.80). ‡ = almost perfect agreement (0.81-1.00).

Abbreviations: AC1 = Agreement Coefficient 1; CC/IT = intercaudate distance to inner-table-width ratio; GCA = global cortical atrophy.

© 2025 Loewen SP et al. *JAMA Network Open*.

eTable 7. Association of Brain Frailty Markers Assessed on NCCT With Excellent Functional Outcome (mRS 0 to 1)

Here, lower odds ratios indicate worse outcomes. Variables significant at *p*<0.05 are bolded; those also significant after Hochberg correction (*p*<0.001) are indicated with an asterisk (*)

**NCCT Brain Frailty Marker**	**OR (95% CI)**	***p* value**	**aOR (95% CI)**	***p* value**
Total Fazekas score (0 = Reference)				
1-2	0.53 (0.41-0.68)	**<0.001***	0.72 (0.49-1.07)	0.11
3-6	0.23 (0.16-0.32)	**<0.001***	0.40 (0.24-0.65)	**<0.001***
Lacunes present	0.59 (0.45-0.77)	**<0.001***	0.75 (0.50-1.11)	0.15
Lacune burden	0.74 (0.65-0.85)	**<0.001***	0.81 (0.67-0.98)	**0.03**
≥1 Chronic infarction	0.52 (0.38-0.73)	**<0.001***	0.70 (0.43-1.14)	0.16
Chronic infarction burden	0.77 (0.66-0.91)	**0.001**	0.86 (0.69-1.07)	0.18
Any lacune or chronic infarct	0.56 (0.45-0.71)	**<0.001***	0.74 (0.52-1.05)	0.10
GCA score (0 = reference)				
1	0.50 (0.39-0.64)	**<0.001***	0.90 (0.63-1.30)	0.58
2-3	0.25 (0.17-0.38)	**<0.001***	0.47 (0.25-0.87)	**0.02**
Subcortical atrophy (CC/IT ratio)	0.92 (0.89-0.94)	**<0.001***	0.99 (0.95-1.03)	0.58
Subcortical atrophy (CC/IT ratio quartiles, Q1 = reference)				
Q2	0.81 (0.58-1.11)	0.19	1.28 (0.78-2.11)	0.33
Q3	0.59 (0.44-0.79)	**0.001**	1.35 (0.83-2.19)	0.23
Q4	0.38 (0.28-0.52)	**<0.001***	0.97 (0.59-1.61)	0.91
Severe cortical or subcortical atrophy	0.49 (0.39-0.61)	**<0.001***	0.80 (0.56-1.14)	0.22
Brain frailty score (0 = reference)				
1	0.60 (0.47-0.77)	**<0.001***	0.87 (0.60-1.27)	0.47
2	0.36 (0.26-0.49)	**<0.001***	0.62 (0.37-1.03)	0.06
3	0.15 (0.09-0.27)	**<0.001***	0.33 (0.16-0.70)	**0.004**

Abbreviations: aOR = adjusted odds ratio; CC/IT = intercaudate distance to inner-table-width ratio; CI = confidence interval; GCA = global cortical atrophy; mRS = modified Rankin scale; NCCT = non-contrast computed tomography.

© 2025 Loewen SP et al. *JAMA Network Open*.

eTable 8. Association of Brain Frailty Markers Assessed on MRI With Excellent Functional Outcome (mRS 0 to 1)

Here, lower odds ratios indicate worse outcomes. Variables significant at $p<0.05$ are bolded; those also significant after Hochberg correction ($p<0.001$) are indicated with an asterisk (*)

MRI Brain Frailty Marker	OR (95% CI)	*p* value	aOR (95% CI)	*p* value
Total Fazekas score (0=Reference)				
1-2	0.60 (0.38-0.95)	**0.03**	0.66 (0.33-1.32)	0.24
3-6	0.29 (0.18-0.48)	**<0.001***	0.33 (0.14-0.74)	**0.008**
Lacunes present	0.62 (0.40-0.94)	**0.03**	0.64 (0.33-1.23)	0.18
Lacune burden	0.77 (0.66-0.90)	**0.001**	0.70 (0.53-0.92)	**0.01**
≥1 Chronic infarction	0.76 (0.43-1.36)	0.36	1.07 (0.44-2.62)	0.88
Chronic infarction burden	0.91 (0.75-1.11)	0.37	1.03 (0.76-1.39)	0.85
Any lacune or chronic infarct	0.70 (0.47-1.02)	0.07	0.81 (0.44-1.47)	0.48
GCA score (0 = reference)				
1	0.66 (0.44-0.97)	**0.03**	0.64 (0.34-1.20)	0.16
2-3	0.29 (0.15-0.53)	**<0.001***	0.43 (0.16-1.15)	0.09
Subcortical atrophy (CC/IT ratio)	0.92 (0.88-0.96)	**0.001**	0.99 (0.92-1.07)	0.80
Subcortical atrophy (CC/IT ratio quartiles, Q1 = reference)				
Q2	0.77 (0.44-1.33)	0.35	0.95 (0.42-2.17)	0.91
Q3	0.56 (0.34-0.92)	**0.02**	1.46 (0.63-3.37)	0.38
Q4	0.40 (0.24-0.67)	**<0.001***	0.90 (0.38-2.13)	0.82
Severe cortical or subcortical atrophy	0.52 (0.36-0.77)	**0.001**	0.91 (0.48-1.70)	0.77
Brain frailty score (0 = reference)				
1	0.69 (0.45-1.07)	0.09	1.23 (0.63-2.43)	0.63
2	0.45 (0.27-0.75)	**0.002**	0.82 (0.34-1.95)	0.53
3	0.31 (0.16-0.59)	**<0.001***	0.35 (0.12-1.01)	0.05
Total EPVS score	0.89 (0.80-0.98)	**0.02**	0.85 (0.73-0.99)	**0.049**
≥1 CMB	0.54 (0.33-0.89)	**0.02**	0.49 (0.22-1.07)	0.07
CMB burden	0.90 (0.80-1.02)	0.11	0.94 (0.83-1.07)	0.36
CSS present	0.32 (0.09-1.14)	0.08	0.40 (0.04-3.82)	0.42
CSS burden (total number of affected sulci)	0.68 (0.39-1.17)	0.16	0.72 (0.28-1.83)	0.48
SVD score (0 = reference)				
1	0.48 (0.30-0.76)	**0.002**	0.43 (0.21-0.89)	**0.02**
2	0.46 (0.27-0.78)	**0.004**	0.63 (0.27-1.48)	0.29
3-4	0.38 (0.21-0.70)	**0.002**	0.28 (0.10-0.76)	**0.01**

Abbreviations: aOR = adjusted odds ratio; CC/IT = intercaudate distance to inner-table-width ratio; CI = confidence interval; CMB = cerebral microbleeds; CSS = cortical superficial siderosis; EPVS = enlarged perivascular spaces; GCA = global cortical atrophy; MRI = magnetic resonance imaging; mRS = modified Rankin scale; SVD = small vessel disease.

© 2025 Loewen SP et al. *JAMA Network Open*.

eTable 9. AUROC for mRS 0 to 1 on NCCT

NCCT Brain Frailty Marker	AUC (95% CI) (adjusted model)
Total Fazekas score	0.73 (0.71-0.76)
Lacunes present	0.71 (0.69-0.74)
Lacune burden	0.71 (0.69-0.74)
≥1 Chronic infarction	0.71 (0.69-0.74)
Chronic infarction burden	0.71 (0.69-0.74)
Any lacune or chronic infarct	0.71 (0.69-0.74)
GCA score	0.72 (0.69-0.74)
Subcortical atrophy (CC/IT ratio)	0.71 (0.69-0.74)
Subcortical atrophy (CC/IT ratio quartiles)	0.72 (0.69-0.74)
Severe cortical or subcortical atrophy	0.71 (0.69-0.74)
Brain frailty score	0.73 (0.70-0.75)

Abbreviations: AUC = area under the curve; CC/IT = intercaudate distance to inner-table-width ratio; GCA = global cortical atrophy; NCCT = non-contrast computed tomography; ROC = receiver operating characteristic.

© 2025 Loewen SP et al. *JAMA Network Open*.

eTable 10. AUROC for mRS 0 to 1 on MRI

MRI Brain Frailty Marker	AUC (95% CI) (adjusted model)
Total Fazekas score	0.69 (0.64-0.73)
Lacunes present	0.67 (0.62-0.72)
Lacune burden	0.68 (0.64-0.73)
≥1 Chronic infarction	0.67 (0.62-0.71)
Chronic infarction burden	0.67 (0.62-0.71)
Any lacune or chronic infarct	0.67 (0.62-0.71)
GCA score	0.68 (0.63-0.73)
Subcortical atrophy (CC/IT ratio)	0.67 (0.63-0.72)
Subcortical atrophy (CC/IT ratio quartiles)	0.68 (0.63-0.73)
Severe cortical or subcortical atrophy	0.67 (0.62-0.72)
Brain frailty score	0.68 (0.63-0.73)
Total EPVS score	0.67 (0.63-0.72)
≥1 CMB	0.66 (0.61-0.71)
CMB burden	0.66 (0.61-0.71)
CSS present	0.67 (0.61-0.71)
CSS burden (total number of affected sulci)	0.66 (0.61-0.71)
SVD score	0.68 (0.63-0.73)

Abbreviations: AUC = area under the curve; CC/IT = intercaudate distance to inner-table-width ratio; CMB = cerebral microbleeds; CSS = cortical superficial siderosis; EPVS = enlarged perivascular spaces; GCA = global cortical atrophy; MRI = magnetic resonance imaging; ROC = receiver operating characteristic; SVD = small vessel disease.

© 2025 Loewen SP et al. *JAMA Network Open*.

eTable 11. Association of Brain Frailty Markers Assessed on NCCT With Functional Outcome (Ordinal mRS)

Here, higher common odds ratios indicate worse outcomes. Variables significant at *p*<0.05 are bolded; those also significant after Hochberg correction (*p*<0.001) are indicated with an asterisk (*)

**NCCT Brain Frailty Marker**	**OR (95% CI)**	***p* value**	**acOR (95% CI)**	***p* value**
Total Fazekas score (0 = reference)				
1-2	2.20 (1.73-2.80)	**<0.001***	1.32 (0.91-1.91)	0.14
3-6	5.18 (4.00-6.72)	**<0.001***	2.80 (1.88-4.16)	**<0.001***
Lacunes present	1.80 (1.43-2.26)	**<0.001***	1.52 (1.09-2.14)	**0.02**
Lacune burden	1.26 (1.14-1.38)	**<0.001***	1.10 (0.97-1.25)	0.14
≥1 Chronic infarction	1.82 (1.41-2.38)	**<0.001***	1.70 (1.14-2.54)	**0.009**
Chronic infarction burden	1.20 (1.07-1.34)	**0.002**	1.16 (0.98-1.36)	0.09
Any lacune or chronic infarct	1.92 (1.56-2.36)	**<0.001***	1.66 (1.22-2.28)	**0.001**
GCA score (0 = reference)				
1	2.43 (1.92-3.05)	**<0.001***	1.37 (0.97-1.92)	0.07
2-3	5.26 (3.81-7.25)	**<0.001***	2.65 (1.63-4.32)	**<0.001***
Subcortical atrophy (CC/IT ratio)	1.08 (1.06-1.11)	**<0.001***	1.01 (0.97-1.04)	0.68
Subcortical atrophy (CC/IT ratio quartiles, Q1 = reference)				
Q2	1.11 (0.79-1.58)	0.38	0.62 (0.37-1.03)	0.06
Q3	2.29 (1.68-3.11)	**<0.001***	0.85 (0.53-1.37)	0.50
Q4	3.36 (2.48-4.55)	**<0.001***	0.98 (0.61-1.47)	0.93
Severe cortical or subcortical atrophy	2.39 (1.95-2.92)	**<0.001***	1.35 (0.98-1.85)	0.07
Brain frailty score (0 = reference)				
1	1.99 (1.56-2.53)	**<0.001***	1.48 (1.02-2.13)	**0.03**
2	3.72 (2.81-4.93)	**<0.001***	2.24 (1.43-3.51)	**<0.001***
3	6.40 (4.44-9.22)	**<0.001***	3.15 (1.87-5.33)	**<0.001***

Abbreviations: acOR = adjusted common odds ratio; CC/IT = intercaudate distance to inner-table-width ratio; CI = confidence interval; GCA = global cortical atrophy; mRS = modified Rankin scale; NCCT = non-contrast computed tomography.

© 2025 Loewen SP et al. *JAMA Network Open*.

eTable 12. Association of Brain Frailty Markers Assessed on MRI With Functional Outcome (Ordinal mRS)

Here, higher common odds ratios indicate worse outcomes. Variables significant at *p*<0.05 are bolded; those also significant after Hochberg correction (*p*<0.001) are indicated with an asterisk (*)

MRI Brain Frailty Marker	OR (95% CI)	*p* value	acOR (95% CI)	*p* value
Total Fazekas score (0 = reference)				
1-2	1.39 (0.81-2.40)	0.24	0.64 (0.29-1.40)	0.27
3-6	3.43 (2.01-5.87)	**<0.001***	1.33 (0.60-2.98)	0.49
Lacunes present	1.89 (1.25-2.86)	**0.003**	1.53 (0.83-2.81)	0.18
Lacune burden	1.18 (1.08-1.28)	**<0.001***	1.17 (1.03-1.32)	**0.02**
≥1 Chronic infarction	1.81 (1.05-3.12)	0.03	1.59 (0.70-3.65)	0.27
Chronic infarction burden	1.10 (0.93-1.32)	0.27	0.94 (0.69-1.27)	0.68
Any lacune or chronic infarct	1.89 (1.28-2.78)	**0.001**	1.66 (0.93-2.96)	0.09
GCA score (0 = reference)				
1	1.82 (1.18-2.80)	**0.007**	1.11 (0.58-2.12)	0.76
2-3	3.39 (1.94-5.88)	**<0.001***	1.78 (0.76-4.17)	0.18
Subcortical atrophy (CC/IT ratio)	1.05 (1.00-1.10)	**0.046**	1.02 (0.95-1.10)	0.52
Subcortical atrophy (CC/IT ratio quartiles, Q1 = reference)				
Q2	1.17 (0.60-2.28)	0.65	0.89 (0.37-2.18)	0.80
Q3	2.71 (1.54-4.80)	**0.001**	0.84 (0.35-2.03)	0.70
Q4	3.70 (2.11-6.47)	**<0.001***	1.52 (0.65-3.58)	0.33
Severe cortical or subcortical atrophy	2.28 (1.57-3.32)	**<0.001***	1.55 (0.84-2.85)	0.16
Brain frailty score (0 = reference)				
1	1.99 (1.23-3.20)	**0.005**	1.38 (0.66-2.86)	0.39
2	2.34 (1.41-3.90)	**0.001**	1.31 (0.56-3.11)	0.53
3	5.09 (2.86-9.05)	**<0.001***	3.31 (1.38-7.98)	**0.008**
Total EPVS score	1.07 (0.96-1.18)	0.21	0.98 (0.85-1.13)	0.78
≥1 CMB	1.90 (1.21-2.97)	**0.005**	1.68 (0.84-3.36)	0.14
CMB burden	1.03 (0.99-1.08)	0.10	1.02 (0.98-1.06)	0.24
CSS present	3.99 (1.60-9.93)	**0.003**	3.64 (0.79-16.8)	0.10
CSS burden (total number of affected sulci)	1.63 (1.13-2.35)	**0.009**	1.62 (0.95-2.77)	0.08
SVD score (0 = reference)				
1	1.90 (1.17-3.10)	**0.01**	1.03 (0.50-2.18)	0.92
2	3.11 (1.88-5.17)	**<0.001***	1.78 (0.77-4.08)	0.18
3-4	2.69 (1.52-4.75)	**<0.001***	1.84 (0.77-4.42)	0.17

Abbreviations: acOR = adjusted common odds ratio; CC/IT = intercaudate distance to inner-table-width ratio; CI = confidence interval; CMB = cerebral microbleeds; CSS = cortical superficial siderosis; EPVS = enlarged perivascular spaces; GCA = global cortical atrophy; MRI = magnetic resonance imaging; mRS = modified Rankin scale; SVD = small vessel disease.

© 2025 Loewen SP et al. *JAMA Network Open*.

eTable 13. Association of Brain Frailty Markers Assessed on NCCT With sICH

Variables significant at *p*<0.05 are bolded; those also significant after Hochberg correction (*p*<0.001) are indicated with an asterisk (*)

NCCT Brain Frailty Marker	OR (95% CI)	*p* value	aOR (95% CI)	*p* value
Total Fazekas score (0 = reference)				
1-2	2.02 (1.02-3.99)	**0.04**	2.51 (0.91-6.96)	0.08
3-6	2.64 (1.32-5.30)	**0.006**	2.21 (0.71-6.87)	0.17
Lacunes present	1.81 (1.01-3.26)	**0.047**	1.42 (0.59-3.42)	0.43
Lacune burden	1.14 (0.91-1.42)	0.27	1.15 (0.87-1.53)	0.33
≥1 Chronic infarction	2.61 (1.40-4.85)	**0.002**	2.73 (1.13-6.59)	**0.03**
Chronic infarction burden	1.25 (0.97-1.62)	0.09	1.32 (0.90-1.92)	0.15
Any lacune or chronic infarct	2.15 (1.23-3.77)	**0.007**	2.18 (0.95-5.00)	0.07
GCA score (0 = reference)				
1	1.33 (0.72-2.47)	0.36	1.95 (0.74-5.11)	0.18
2-3	1.80 (0.81-4.01)	0.15	1.90 (0.51-7.07)	0.34
Subcortical atrophy (CC/IT ratio)	1.02 (0.99-1.05)	0.16	1.07 (0.96-1.18)	0.23
Subcortical atrophy (CC/IT ratio quartiles, Q1 = reference)				
Q2	2.12 (0.77-5.81)	0.14	0.92 (0.18-4.75)	0.92
Q3	1.74 (0.66-4.58)	0.26	1.64 (0.41-6.59)	0.48
Q4	2.11 (0.84-5.31)	0.11	1.59 (0.40-6.35)	0.51
Severe cortical or subcortical atrophy	1.34 (0.76-2.35)	0.31	1.40 (0.59-3.29)	0.44
Brain frailty score (0 = reference)				
1	1.71 (0.81-3.58)	0.16	1.27 (0.43-3.74)	0.67
2	2.79 (1.39-5.61)	**0.005**	1.79 (0.53-6.06)	0.35
3	2.47 (0.92-6.61)	0.07	2.97 (0.85-10.45)	0.09

Abbreviations: aOR = adjusted odds ratio; CC/IT = intercaudate distance to inner-table-width ratio; CI = confidence interval; GCA = global cortical atrophy; mRS = modified Rankin scale; NCCT = non-contrast computed tomography; sICH = symptomatic intracerebral haemorrhage.

© 2025 Loewen SP et al. *JAMA Network Open*.

eTable 14. Association of Brain Frailty Markers Assessed on NCCT With Radiographic ICH

Variables significant at *p*<0.05 are bolded; those also significant after Hochberg correction (*p*<0.001) are indicated with an asterisk (*)

**NCCT Brain Frailty Marker**	**OR (95% CI)**	***p* value**	**aOR (95% CI)**	***p* value**
Total Fazekas score (0 = reference)				
1-2	1.07 (0.79-1.45)	0.66	1.04 (0.67-1.63)	0.85
3-6	1.09 (0.79-1.52)	0.60	0.78 (0.47-1.30)	0.34
Lacunes present	1.04 (0.77-1.41)	0.78	1.01 (0.66-1.55)	0.95
Lacune burden	0.99 (0.88-1.13)	0.93	1.00 (0.86-1.18)	0.96
≥1 Chronic infarction	1.40 (0.99-1.98)	0.05	0.92 (0.55-1.53)	0.74
Chronic infarction burden	1.16 (1.00-1.34)	**0.04**	1.03 (0.83-1.28)	0.77
Any lacune or chronic infarct	1.14 (0.87-1.49)	0.33	0.96 (0.65-1.42)	0.83
GCA score (0 = reference)				
1	1.32 (0.99-1.78)	0.06	1.27 (0.83-1.93)	0.28
2-3	1.40 (0.92-2.10)	0.11	0.77 (0.40-1.51)	0.45
Subcortical atrophy (CC/IT ratio)	1.01 (0.99-1.04)	0.27	0.96 (0.91-1.01)	0.10
Subcortical atrophy (CC/IT ratio quartiles, Q1 = reference)				
Q2	0.91 (0.60-1.39)	0.67	0.72 (0.39-1.31)	0.29
Q3	1.07 (0.73-1.55)	0.73	0.92 (0.53-1.62)	0.78
Q4	1.01 (0.70-1.47)	0.94	0.61 (0.34-1.09)	0.10
Severe cortical or subcortical atrophy	1.06 (0.81-1.38)	0.67	0.78 (0.52-1.17)	0.24
Brain frailty score (0 = reference)				
1	1.21 (0.90-1.64)	0.21	1.03 (0.67-1.60)	0.88
2	1.09 (0.76-1.56)	0.64	0.64 (0.35-1.14)	0.13
3	1.23 (0.76-1.97)	0.40	0.85 (0.43-1.66)	0.63

Abbreviations: aOR = adjusted odds ratio; CC/IT = intercaudate distance to inner-table-width ratio; CI = confidence interval; GCA = global cortical atrophy; ICH = intracerebral haemorrhage; mRS = modified Rankin scale; NCCT = non-contrast computed tomography.

© 2025 Loewen SP et al. *JAMA Network Open*.

eTable 15. Association of Brain Frailty Markers Assessed on NCCT With ICH Severity (Ordinal Scale According to the Heidelberg Classification System)

Variables significant at *p*<0.05 are bolded; those also significant after Hochberg correction (*p*<0.001) are indicated with an asterisk (*)

**NCCT Brain Frailty Marker**	**OR (95% CI)**	***p* value**	**acOR (95% CI)**	***p* value**
Total Fazekas score (0 = reference)				
1-2	1.05 (0.77-1.45)	0.75	1.04 (0.65-1.67)	0.87
3-6	1.02 (0.71-1.45)	0.93	0.90 (0.53-1.55)	0.71
Lacunes present	1.08 (0.79-1.49)	0.62	1.00 (0.64-1.58)	0.99
Lacune burden	1.01 (0.89-1.15)	0.86	1.02 (0.86-1.20)	0.85
≥1 Chronic infarction	1.27 (0.87-1.84)	0.22	0.82 (0.46-1.45)	0.49
Chronic infarction burden	1.05 (0.89-1.23)	0.57	0.86 (0.66-1.12)	0.27
Any lacune or chronic infarct	1.14 (0.86-1.52)	0.36	0.97 (0.64-1.48)	0.90
GCA score (0 = reference)				
1	1.21 (0.89-1.65)	0.23	1.19 (0.76-1.86)	0.45
2-3	1.18 (0.76-1.84)	0.46	0.71 (0.34-1.47)	0.35
Subcortical atrophy (CC/IT ratio)	1.01 (0.99-1.03)	0.38	0.97 (0.92-1.02)	0.20
Subcortical atrophy (CC/IT ratio quartiles, Q1 = reference)				
Q2	0.84 (0.54-1.31)	0.45	0.71 (0.38-1.32)	0.28
Q3	1.01 (0.68-1.49)	0.98	0.86 (0.48-1.56)	0.63
Q4	0.90 (0.61-1.34)	0.62	0.64 (0.35-1.17)	0.15
Severe cortical or subcortical atrophy	0.96 (0.72-1.28)	0.77	0.79 (0.51-1.21)	0.28
Brain frailty score (0 = reference)				
1	1.20 (0.88-1.65)	0.26	0.99 (0.63-1.58)	0.98
2	1.02 (0.69-1.50)	0.94	0.67 (0.36-1.24)	0.20
3	1.09 (0.64-1.84)	0.76	0.96 (0.47-1.97)	0.91

Abbreviations: acOR = adjusted common odds ratio; CC/IT = intercaudate distance to inner-table-width ratio; CI = confidence interval; GCA = global cortical atrophy; ICH = intracerebral haemorrhage; mRS = modified Rankin scale; NCCT = non-contrast computed tomography.

© 2025 Loewen SP et al. *JAMA Network Open*.

eTable 16. Association of Brain Frailty Markers Assessed on NCCT With Mortality (mRS 6)

Variables significant at *p*<0.05 are bolded; those also significant after Hochberg correction (*p*<0.001) are indicated with an asterisk (*)

**NCCT Brain Frailty Marker**	**OR (95% CI)**	***p* value**	**aOR (95% CI)**	***p* value**
Total Fazekas score (0 = reference)				
1-2	1.95 (1.37-2.78)	**<0.001***	0.93 (0.53-1.61)	0.79
3-6	3.84 (2.71-5.44)	**<0.001***	1.75 (1.02-3.00)	**0.04**
Lacunes present	1.63 (1.19-2.23)	**0.002**	1.40 (0.88-2.25)	0.16
Lacune burden	1.15 (1.01-1.30)	**0.03**	1.01 (0.84-1.21)	0.90
≥1 Chronic infarction	1.50 (1.05-2.16)	**0.03**	1.38 (0.80-2.39)	0.25
Chronic infarction burden	1.11 (0.95-1.31)	0.19	1.19 (0.94-1.51)	0.15
Any lacune or chronic infarct	1.67 (1.25-2.22)	**<0.001***	1.41 (0.90-2.19)	0.13
GCA score (0 = reference)				
1	2.75 (1.95-3.89)	**<0.001***	1.53 (0.91-2.57)	
2-3	5.39 (3.55-8.20)	**<0.001***	2.88 (1.51-5.49)	**0.001**
Subcortical atrophy (CC/IT ratio)	1.05 (1.02-1.08)	**0.002**	0.98 (0.92-1.03)	0.41
Subcortical atrophy (CC/IT ratio quartiles, Q1 = reference)				
Q2	1.10 (0.65-1.88)	0.71	0.58 (0.27-1.25)	0.16
Q3	1.85 (1.17-2.92)	**0.008**	0.67 (0.34-1.34)	0.26
Q4	2.59 (1.66-4.04)	**<0.001***	0.60 (0.30-1.19)	0.15
Severe cortical or subcortical atrophy	2.19 (1.65-2.92)	**<0.001***	1.07 (0.68-1.67)	0.78
Brain frailty score (0 = reference)				
1	1.84 (1.27-2.66)	**0.001**	1.27 (0.73-2.19)	0.40
2	3.42 (2.31-5.06)	**<0.001***	1.37 (0.72-2.63)	0.34
3	4.36 (2.70-7.02)	**<0.001***	2.17 (1.09-4.33)	**0.03**

Abbreviations: aOR = adjusted odds ratio; CC/IT = intercaudate distance to inner-table-width ratio; CI = confidence interval; GCA = global cortical atrophy; mRS = modified Rankin scale; NCCT = non-contrast computed tomography.

© 2025 Loewen SP et al. *JAMA Network Open*.

eTable 17. Association of Brain Frailty Markers Assessed on MRI With Symptomatic ICH

Variables significant at *p*<0.05 are bolded; those also significant after Hochberg correction (*p*<0.001) are indicated with an asterisk (*)

**MRI Brain Frailty Marker**	**OR (95% CI)**	***p* value**	**aOR (95% CI)**	***p* value**
Total Fazekas score (0 = reference)				
1-2	0.39 (0.06-2.36)	0.30	0.89 (0.05-15.79)	0.93
3-6	0.95 (0.21-4.32)	0.95	4.59 (0.33-63.89)	0.26
Lacunes present	0.36 (0.04-2.91)	0.34	0.85 (0.09-8.47)	0.89
Lacune burden	0.46 (0.10-2.22)	0.33	0.66 (0.15-2.85)	0.58
≥1 Chronic infarction	0.96 (0.12-7.80)	0.97	2.40 (0.22-26.60)	0.48
Chronic infarction burden	0.73 (0.20-2.58)	0.62	0.99 (0.26-3.78)	0.99
Any lacune or chronic infarct	0.56 (0.12-2.74)	0.48	1.62 (0.25-10.71)	0.62
GCA score (0 = reference)				
1-3	0.53 (0.13-2.25)	0.39	0.56 (0.08-4.08)	0.57
Subcortical atrophy (CC/IT ratio)	0.73 (0.17-3.10)	0.76	0.96 (0.75-1.22)	0.75
Subcortical atrophy (CC/IT ratio quartiles, Q1 = reference)				
Q2	1.20 (0.17-8.67)	0.86	1.00 (0.06-17.55)	0.99
Q3	1.95 (0.37-10.22)	0.43	3.86 (0.35-43.17)	0.27
Q4	0.38 (0.03-4.20)	0.43	0.86 (0.04-16.96)	0.92
Severe cortical or subcortical atrophy	0.20 (0.03-1.61)	0.13	0.49 (0.05-5.19)	0.56
Brain frailty score (0 = reference)				
1	0.92 (0.22-3.92)	0.91	2.19 (0.31-15.54)	0.43
2-3	0.53 (0.10-2.75)	0.45	0.94 (0.07-13.51)	0.96
Total EPVS score	0.54 (0.28-1.06)	0.08	0.20 (0.03-1.37)	0.10
CMB burden	1.17 (1.05-1.30)	**0.005**	1.20 (1.01-1.43)	**0.04**
CSS present	3.55 (0.42-29.95)	0.25	14.83 (1.19-184.4)	**0.04**
CSS burden (total number of affected sulci)	1.58 (0.84-2.96)	0.16	3.30 (1.24-8.77)	**0.02**
SVD score (0 = reference)				
1	3.06 (0.67-13.91)	0.15	10.44 (0.85-128.5)	0.07
2	2.01 (0.33-12.23)	0.45	18.91 (1.25-286.9)	**0.03**
3-4	1.36 (0.14-13.33)	0.79	5.75 (0.38-87.64)	0.21

Abbreviations: aOR = adjusted odds ratio; CC/IT = intercaudate distance to inner-table-width ratio; CI = confidence interval; CMB = cerebral microbleeds; CSS = cortical superficial siderosis; EPVS = enlarged perivascular spaces; GCA = global cortical atrophy; MRI = magnetic resonance imaging; mRS = modified Rankin scale, sICH = symptomatic intracerebral haemorrhage; SVD = small vessel disease.

© 2025 Loewen SP et al. *JAMA Network Open*.

eTable 18. Association of Brain Frailty Markers Assessed on MRI With Radiographic ICH

Variables significant at *p*<0.05 are bolded; those also significant after Hochberg correction (*p*<0.001) are indicated with an asterisk (*)

MRI Brain Frailty Marker	OR (95% CI)	*p* value	aOR (95% CI)	*p* value
Total Fazekas score (0 = reference)				
1-2	0.59 (0.35-0.99)	**0.046**	0.47 (0.22-0.98)	**0.04**
3-6	0.81 (0.48-1.36)	0.42	0.45 (0.20-1.02)	0.06
Lacunes present	0.97 (0.60-1.56)	0.89	0.84 (0.42-1.65)	0.60
Lacune burden	1.07 (0.97-1.18)	0.20	0.96 (0.81-1.13)	0.60
≥1 Chronic infarction	1.26 (0.68-2.33)	0.47	0.89 (0.36-2.22)	0.81
Chronic infarction burden	1.12 (0.92-1.36)	0.24	1.06 (0.78-1.43)	0.72
Any lacune or chronic infarct	1.02 (0.66-1.58)	0.92	0.73 (0.39-1.39)	0.34
GCA score (0 = reference)				
1	1.16 (0.74-1.82)	0.52	1.04 (0.54-2.01)	0.90
2-3	1.18 (0.64-2.17)	0.61	0.58 (0.21-1.60)	0.29
Subcortical atrophy (CC/IT ratio)	1.02 (0.99-1.05)	0.23	0.94 (0.86-1.02)	0.13
Subcortical atrophy (CC/IT ratio quartiles, Q1 = reference)				
Q2	0.86 (0.44-1.65)	0.64	0.60 (0.25-1.44)	0.25
Q3	1.28 (0.73-2.26)	0.39	0.85 (0.36-1.98)	0.70
Q4	0.96 (0.53-1.72)	0.88	0.54 (0.22-1.31)	0.17
Severe cortical or subcortical atrophy	0.98 (0.63-1.51)	0.91	0.66 (0.33-1.30)	0.23
Brain frailty score (0 = reference)				
1	1.18 (0.72-1.93)	0.52	1.13 (0.56-2.29)	0.73
2	1.07 (0.61-1.87)	0.82	0.76 (0.31-1.88)	0.55
3	1.06 (0.54-2.08)	0.86	0.50 (0.18-1.44)	0.20
Total EPVS score	0.90 (0.79-1.02)	0.09	0.82 (0.69-0.97)	**0.02**
≥1 CMB	1.69 (1.03-2.76)	**0.04**	1.92 (0.93-3.96)	0.08
CMB burden	1.10 (1.00-1.21)	**0.04**	1.09 (0.98-1.21)	0.12
CSS present	8.18 (2.55-26.3)	**<0.001***	3.82 (0.66-22.0)	0.13
CSS burden (total number of affected sulci)	2.99 (1.48-6.06)	**0.002**	1.98 (0.77-5.06)	0.16
SVD score (0 = reference)				
1	1.37 (0.83-2.28)	0.22	1.22 (0.59-2.52)	0.59
2	1.21 (0.69-2.15)	0.50	0.78 (0.31-1.93)	0.59
3-4	1.05 (0.54-2.03)	0.89	0.79 (0.29-2.12)	0.64

Abbreviations: aOR = adjusted odds ratio; CC/IT = intercaudate distance to inner-table-width ratio; CI = confidence interval; CMB = cerebral microbleeds; CSS = cortical superficial siderosis; EPVS = enlarged perivascular spaces; GCA = global cortical atrophy; ICH = intracerebral haemorrhage; MRI = magnetic resonance imaging; mRS = modified Rankin scale; SVD = small vessel disease.

© 2025 Loewen SP et al. *JAMA Network Open*.

eTable 19. Association of Brain Frailty Markers Assessed on MRI With ICH Severity
Variables significant at *p*<0.05 are bolded; those also significant after Hochberg correction (*p*<0.001) are indicated with an asterisk (*)

MRI Brain Frailty Marker	OR (95% CI)	*p* value	acOR (95% CI)	*p* value
Total Fazekas score (0 = reference)				
1-2	0.58 (0.34-0.99)	**0.04**	0.49 (0.23-1.04)	0.06
3-6	0.79 (0.47-1.34)	0.38	0.51 (0.22-1.18)	0.12
Lacunes present	0.84 (0.51-1.38)	0.49	0.67 (0.33-1.36)	0.26
Lacune burden	1.02 (0.93-1.13)	0.66	0.93 (0.79-1.10)	0.40
≥1 Chronic infarction	0.97 (0.50-1.87)	0.93	0.67 (0.25-1.76)	0.41
Chronic infarction burden	0.98 (0.79-1.22)	0.85	0.78 (0.51-1.21)	0.27
Any lacune or chronic infarct	0.92 (0.58-1.44)	0.71	0.65 (0.34-1.26)	0.20
GCA score (0 = reference)				
1	1.14 (0.73-1.79)	0.57	1.18 (0.60-2.30)	0.63
2-3	1.13 (0.60-2.10)	0.71	0.58 (0.21-1.64)	0.31
Subcortical atrophy (CC/IT ratio)	1.01 (0.99-1.04)	0.33	0.92 (0.84-0.99)	**0.04**
Subcortical atrophy (CC/IT ratio quartiles, Q1 = reference)				
Q2	0.78 (0.41-1.48)	0.44	0.50 (0.21-1.19)	0.12
Q3	1.12 (0.64-1.96)	0.70	0.64 (0.27-1.52)	0.31
Q4	0.77 (0.43-1.37)	0.38	0.37 (0.15-0.91)	**0.03**
Severe cortical or subcortical atrophy	0.89 (0.58-1.37)	0.59	0.57 (0.29-1.11)	0.10
Brain frailty score (0 = reference)				
1	1.22 (0.75-2.01)	0.42	1.12 (0.55-2.28)	0.75
2	0.89 (0.50-1.61)	0.71	0.67 (0.26-1.75)	0.42
3	0.98 (0.50-1.92)	0.95	0.44 (0.15-1.28)	0.13
Total EPVS score	0.89 (0.79-0.99)	**0.04**	0.84 (0.71-1.00)	0.05
≥1 CMB	1.50 (0.90-2.50)	0.12	1.85 (0.88-3.87)	0.10
CMB burden	1.09 (1.02-1.15)	**0.006**	1.09 (1.02-1.16)	**0.008**
CSS present	4.11 (1.52-11.13)	**0.005**	1.06 (0.19-5.97)	0.95
CSS burden (total number of affected sulci)	1.68 (1.05-2.69)	**0.03**	0.95 (0.51-1.76)	0.86
SVD score (0 = reference)				
1	1.46 (0.88-2.42)	0.14	1.41 (0.68-2.93)	0.25
2	0.88 (0.47-1.64)	0.69	0.47 (0.16-1.38)	0.17
3-4	1.02 (0.53-1.98)	0.95	0.87 (0.32-2.33)	0.78

Abbreviations: acOR = adjusted common odds ratio; CC/IT = intercaudate distance to inner-table-width ratio; CI = confidence interval; CMB = cerebral microbleeds; CSS = cortical superficial siderosis; EPVS = enlarged perivascular spaces; GCA = global cortical atrophy; ICH = intracerebral haemorrhage; MRI = magnetic resonance imaging; mRS = modified Rankin scale; SVD = small vessel disease.

© 2025 Loewen SP et al. *JAMA Network Open*.

eTable 20. Association of Brain Frailty Markers Assessed on MRI With Mortality (mRS 6)

Variables significant at *p*<0.05 are bolded; those also significant after Hochberg correction (*p*<0.001) are indicated with an asterisk (*)

MRI Brain Frailty Marker	OR (95% CI)	*p* value	aOR (95% CI)	*p* value
Total Fazekas score (0 = reference)				
1-2	1.38 (0.52-3.70)	0.52	0.85 (0.23-3.12)	0.80
3-6	1.94 (0.74-5.12)	0.18	0.87 (0.22-3.39)	0.84
Lacunes present	1.71 (0.84-3.49)	0.14	1.37 (0.52-3.64)	0.52
Lacune burden	1.13 (1.01-1.28)	**0.03**	1.09 (0.92-1.29)	0.30
≥1 Chronic infarction	2.36 (1.02-5.48)	**0.045**	2.21 (0.71-6.94)	0.17
Chronic infarction burden	1.16 (0.88-1.51)	0.29	1.10 (0.77-1.58)	0.59
Any lacune or chronic infarct	1.63 (0.82-3.24)	0.16	1.61 (0.63-4.13)	0.32
GCA score (0 = reference)				
1	2.17 (0.97-4.87)	0.06	1.09 (0.36-3.32)	0.88
2-3	3.24 (1.26-8.33)	**0.02**	1.87 (0.49-7.10)	0.36
Subcortical atrophy (CC/IT ratio)	1.02 (0.98-1.05)	0.31	0.98 (0.87-1.11)	0.78
Subcortical atrophy (CC/IT ratio quartiles, Q1 = reference)				
Q2	1.59 (0.35-7.28)	0.55	1.31 (0.27-6.44)	0.74
Q3	3.83 (1.07-13.70)	**0.04**	1.39 (0.29-6.71)	0.68
Q4	4.05 (1.14-14.35)	**0.03**	1.63 (0.35-7.62)	0.54
Severe cortical or subcortical atrophy	2.18 (1.10-4.32)	**0.03**	1.45 (0.53-3.97)	0.47
Brain frailty score (0 = reference)				
1	2.39 (0.95-6.00)	0.07	1.00 (0.28-3.59)	0.99
2	2.51 (0.94-6.72)	0.07	1.11 (0.27-4.55)	0.88
3	3.26 (1.13-9.42)	**0.03**	1.90 (0.48-7.48)	0.36
Total EPVS score	1.08 (0.91-1.27)	0.37	1.02 (0.81-1.27)	0.90
≥1 CMB	0.98 (0.39-2.47)	0.97	0.54 (0.14-2.04)	0.37
CMB burden	1.01 (0.93-1.09)	0.89	0.96 (0.80-1.15)	0.67
CSS present	3.65 (0.97-13.65)	0.06	4.06 (0.64-25.77)	0.14
CSS burden (total number of affected sulci)	1.60 (1.00-2.54)	**0.049**	1.69 (0.91-3.13)	0.09
SVD score (0 = reference)				
1	1.57 (0.65-3.80)	0.32	0.40 (0.10-1.65)	0.21
2	1.89 (0.75-4.73)	0.18	0.80 (0.21-2.99)	0.74
3-4	1.94 (0.71-5.34)	0.20	0.84 (0.22-3.28)	0.80

Abbreviations: aOR = adjusted odds ratio; CC/IT = intercaudate distance to inner-table-width ratio; CI = confidence interval; CMB = cerebral microbleeds; CSS = cortical superficial siderosis; EPVS = enlarged perivascular spaces; GCA = global cortical atrophy; MRI = magnetic resonance imaging; mRS = modified Rankin scale; SVD = small vessel disease.

© 2025 Loewen SP et al. *JAMA Network Open*.

eReferences

1. Pasquier F, Leys D, Weerts JGE, Mounier-Vehier F, Barkhof F, Scheltens P. Inter-and Intraobserver Reproducibility of Cerebral Atrophy Assessment on MRI Scans with Hemispheric Infarcts. *Eur Neurol*. 1996;36(5):268-272.

2. Lee SH, Oh CW, Han JH, et al. The effect of brain atrophy on outcome after a large cerebral infarction. *J Neurol Neurosurg Psychiatry*. 2010;81(12):1316-1321.

3. Duering M, Biessels GJ, Brodtmann A, et al. Neuroimaging standards for research into small vessel disease—advances since 2013. *Lancet Neurol*. 2023;22(7):602-618.

4. Fazekas F, Chawluk JB, Alavi A, Hurtig HI, Zimmerman RA. MR signal abnormalities at 1.5 T in Alzheimer's dementia and normal aging. *AJR Am J Roentgenol*. 1987;149(2):351-356.

5. Wattjes MP, Henneman WJP, van der Flier WM, et al. Diagnostic imaging of patients in a memory clinic: comparison of MR imaging and 64-detector row CT. *Radiology*. 2009;253(1):174-183.

6. Ferguson KJ, Cvoro V, MacLullich AMJ, et al. Visual Rating Scales of White Matter Hyperintensities and Atrophy: Comparison of Computed Tomography and Magnetic Resonance Imaging. *J Stroke Cerebrovasc Dis*. 2018;27(7):1815-1821.

7. Betzner W, Singh N, Alhabli I, et al. Abstract WMP69: Brain Atrophy and White Matter Disease Agreement on NCCT and MRI in Ischemic Stroke. *Stroke*. 2024;55(Suppl_1):AWMP69-AWMP69.

8. Cordonnier C, Potter GM, Jackson CA, et al. Improving Interrater Agreement About Brain Microbleeds: Development of the Brain Observer MicroBleed Scale (BOMBS). *Stroke*. 2009;40(1):94-99.

9. Potter GM, Chappell FM, Morris Z, Wardlaw JM. Cerebral perivascular spaces visible on magnetic resonance imaging: development of a qualitative rating scale and its observer reliability. *Cerebrovasc Dis*. 2015;39(3-4):224-231.

10. Staals J, Makin SDJ, Doubal FN, Dennis MS, Wardlaw JM. Stroke subtype, vascular risk factors, and total MRI brain small-vessel disease burden. *Neurology*. 2014;83(14):1228-1234.

11. Appleton JP, Woodhouse LJ, Adami A, et al. Imaging markers of small vessel disease and brain frailty, and outcomes in acute stroke. *Neurology*. 2020;94(5):e439-e452.

© 2025 Loewen SP et al. *JAMA Network Open*.
