# Supplementary material for: Brain Frailty and Functional Outcomes After Thrombolysis for Acute Ischemic Stroke
Source: JAMA Netw Open. 2025 Sep 30;8(9):e2534941. doi: 10.1001/jamanetworkopen.2025.34941 (PMC12485648; doi:10.1001/jamanetworkopen.2025.34941)
Supplement: Supplement 2. — Data Sharing Statement [file jamanetwopen-e2534941-s002.pdf]

Data Sharing Statement

Loewen. Brain Frailty and Functional Outcomes After Thrombolysis for Acute Ischemic Stroke. *JAMA Netw Open*. Published September 30, 2025. doi:10.1001/jamanetworkopen.2025.34941

Data
Data available: Yes
Data types: Other (please specify)
Additional Information: Data from this study are currently not publicly available. We will share anonymized data upon reasonable request from any qualified investigator after clearance by the local ethics committee. A request for access to the data can be made by sending an email together with a research plan to aganesh@ucalgary.ca.
How to access data: Data from this study are currently not publicly available. We will share anonymized data upon reasonable request from any qualified investigator after clearance by the local ethics committee. A request for access to the data can be made by sending an email together with a research plan to aganesh@ucalgary.ca.
When available: With publication

Supporting Documents
Document types: None

Additional Information
Who can access the data: Qualified investigator after clearance by the local ethics committee
Types of analyses: Any purpose
Mechanisms of data availability: Without investigator support and after approval of a proposal and a signed data access agreement
Any additional restrictions: Canada law, law of participating centers and regulations of respective ethics committees
